# Modelling the Emergence and Dynamics of Perceptual Organisation in Auditory Streaming

**DOI:** 10.1371/journal.pcbi.1002925

**Published:** 2013-03-14

**Authors:** Robert W. Mill, Tamás M. Bőhm, Alexandra Bendixen, István Winkler, Susan L. Denham

**Affiliations:** 1 MRC Institute of Hearing Research, Nottingham, United Kingdom; 2 Institute of Cognitive Neuroscience and Psychology, Research Centre for Natural Sciences, MTA, Budapest, Hungary; 3 Department of Telecommunications and Media Informatics, Budapest University of Technology and Economics, Budapest, Hungary; 4 Institute for Psychology, University of Leipzig, Leipzig, Germany; 5 Institute for Psychology, University of Szeged, Szeged, Hungary; 6 Cognition Institute and School of Psychology, University of Plymouth, Plymouth, United Kingdom; Indiana University, United States of America

## Abstract

Many sound sources can only be recognised from the pattern of sounds they emit, and not from the individual sound events that make up their emission sequences. Auditory scene analysis addresses the difficult task of interpreting the sound world in terms of an unknown number of discrete sound sources (causes) with possibly overlapping signals, and therefore of associating each event with the appropriate source. There are potentially many different ways in which incoming events can be assigned to different causes, which means that the auditory system has to choose between them. This problem has been studied for many years using the auditory streaming paradigm, and recently it has become apparent that instead of making one fixed perceptual decision, given sufficient time, auditory perception switches back and forth between the alternatives—a phenomenon known as perceptual bi- or multi-stability. We propose a new model of auditory scene analysis at the core of which is a process that seeks to discover predictable patterns in the ongoing sound sequence. Representations of predictable fragments are created on the fly, and are maintained, strengthened or weakened on the basis of their predictive success, and conflict with other representations. Auditory perceptual organisation emerges spontaneously from the nature of the competition between these representations. We present detailed comparisons between the model simulations and data from an auditory streaming experiment, and show that the model accounts for many important findings, including: the emergence of, and switching between, alternative organisations; the influence of stimulus parameters on perceptual dominance, switching rate and perceptual phase durations; and the build-up of auditory streaming. The principal contribution of the model is to show that a two-stage process of pattern discovery and competition between incompatible patterns can account for both the contents (perceptual organisations) and the dynamics of human perception in auditory streaming.

## Author Summary

The sound waves produced by objects in the environment mix together before reaching the ears. Before we can make sense of an auditory scene, our brains must solve the puzzle of how to disassemble the sound waveform into groupings that correspond to the original source signals. How is this feat accomplished? We propose that the auditory system continually scans the structure of incoming signals in search of clues to indicate which pieces belong together. For instance, sound events may belong together if they have similar features, or form part of a clear temporal pattern. However this process is complicated by lack of knowledge of future events and the many possible ways in which even a simple sound sequence can be decomposed. The biological solution is multistability: one possible interpretation of a sound is perceived initially, which then gives way to another interpretation, and so on. We propose a model of auditory multistability, in which fragmental descriptions of the signal compete and cooperate to explain the sound scene. We demonstrate, using simplified experimental stimuli, that the model can account for both the contents (perceptual organisations) and the dynamics of human perception in auditory streaming.

## Introduction

Ecologically valid acoustic signals exhibit structure on multiple time scales. For example, the structure in an orchestral symphony ranges from the sub-millisecond time range (e.g., vibrations of strings) through layers of melodic and rhythmic patterns measurable in seconds, to the overall musical composition that may last for tens of minutes. Such complex scenarios require the interpretation of multiscale articulate patterns, demanding that the brain draw on a wide repertoire of decoding strategies. The overall perceptual task of analysing an entire sound mixture into meaningful elements, or auditory objects [1], [2], is termed *auditory scene analysis* [3]. Intermediate time scales spanning a few hundred milliseconds to a few seconds serve a special role, as they form our immediate subjective experience of incoming sounds [4], [5], providing the basis for working memory [6], [7]. Patterns emerging within this time range pose a challenge to the perceptual system, as detecting and representing them often involves connecting sounds separated by silent periods. Here we propose a new model for the perceptual encoding of sequences of discrete sounds presented at these intermediate rates. Our model focuses on processing ambiguous input in a manner similar to human listeners, because such stimulus configurations provide insights into the hidden mechanics of perceptual processes [8]. The model accounts for the contents and dynamics of perceptual awareness in auditory streaming experiments, and provides a new theoretical interpretation of the perceptual strategies underlying our ability to make timely yet flexible perceptual decisions.

Sequences on the intermediate time scale addressed here are ubiquitous in both natural and experimental settings. For example, the sound of a solitary footstep is perceived as a tap, but a regular procession of footsteps evokes the sense of a persistent and readily recognisable source [9]. How, then, does the brain group these footsteps? To explore all possible groupings is to invite a combinatorial explosion (twelve footsteps can be grouped in over a million ways); yet to assign all footsteps to a single source is to neglect the possibility of two or more walkers. Naturally, in trading economy and flexibility, the brain favours neither extreme; instead, it groups sounds on the basis of their similarity and by searching for ecologically valid patterns into which they could fall [3], [10]–[14] (cf. the Gestalt grouping principles [15]).

For certain stimuli, a degree of ambiguity remains even after ecologically unlikely interpretations have been dismissed. This seems to be especially true for stimuli which have been stripped of the disambiguating cues that are generally present, such as depth and shading (a wireframe Necker cube) or a distinctive timbre (a sequence of pure tones). Sensory input of this sort gives rise to *multistable perception* [16], [17], which is characterised in conscious experience by the spontaneous switching of interpretations from one alternative to another. In vision research, *binocular rivalry*—a particular instance of perceptual bistability, in which the two eyes, when presented with disparate images, compete for dominance [18]—has been the scene of particularly intensive analysis and modelling efforts (e.g. [19]–[23], for reviews, see [24], [25]). The dynamics that govern switching are reminiscent of those that govern the alternation of a noisy phase particle between two attractors in an energy landscape [20]. In terms of perception, attractors are quasi-stable states of the system, each of which is assumed to correspond to a discrete *perceptual state*. An attractor can be stable for some observable period of time, thereby modelling a *perceptual phase*, the time during which a particular percept is experienced. Phenomenological models based on the concept of attractor dynamics are thus able to reproduce many aspects of the data and do so in a biologically plausible fashion (e.g. [21], [23]). Recent evidence suggests that multistable perception is governed by similar processes in the auditory and visual modality [26], [27]. Notably, perceptual phase durations tend to conform to a gamma- or log-normal-like distribution [23], [28], [29], and successive durations are only weakly correlated [28], [30], [31]. However, to our knowledge the attractor dynamics perspective has not yet been applied to multistable perceptual phenomena in the auditory domain.

Auditory perceptual bi-/multistability has been most extensively studied using the streaming paradigm [26], [32]–[35], introduced by van Noorden [30], in which sound sequences are presented in the pattern ABA−ABA−…, where “−” denotes a silent interval equal in duration to one of the sounds [3], [11], [30]. The two most stable percepts are labelled *integrated* and *segregated*, depending on whether one hears the “A” and “B” sounds as arising from a single source or two separate sources, respectively. The traditional assumption is that the default perception is that of integration, and that segregation emerges through differential suppression [36]–[40] (i.e., some of the neurons which initially respond to both “A” and “B” subsequently come to respond to a much lesser extent to one set of sounds than to the other). However, a differential suppression based view of streaming cannot, of itself, explain bistability; it does not account for ongoing switching between qualitatively different perceptual organisations. Therefore, on the basis of the similarities between auditory and visual perceptual bistability, we propose a model that provides an attractor dynamics account of auditory streaming.

If this approach is adopted, the question then is: what are the attractors, and how are they formed? Models of visual bistability tend to assume that the attractors (the possible percepts) are known *a priori* (e.g. [20], [22], [23], see [20] for a generalisation to an arbitrary number of predefined attractors). This simplification may be reasonable where the attractors are predetermined by organic correspondence, e.g., the two eyes in binocular rivalry. However, assuming *a priori* the identity of fixed percepts for sound sequences would neglect the fact that most streaming sequences evoke quite varied percepts in listeners, and thus the attractors, and even how many attractors there are, may be idiosyncratic (e.g. see [41]). Therefore, we propose a model that goes beyond previous models in vision and shapes the attractor landscape dynamically.

### Principles of modelling

**Figure 1:**
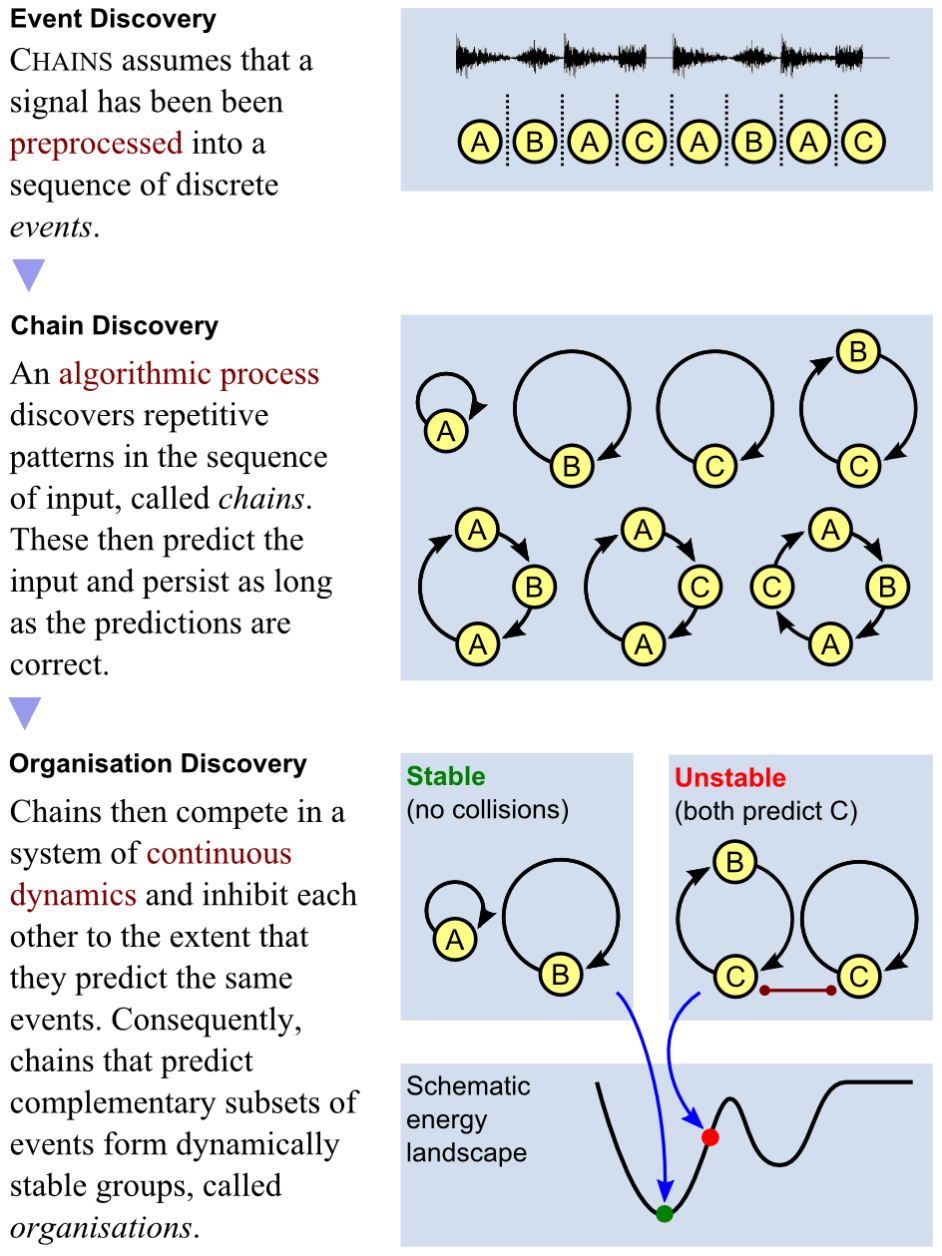
Model overview.

Conceptually, the model consists of two stages: the first is concerned with the discovery of the attractors, and the second with the form of the competition between them. Together they account for the nature of perceptual awareness. However, it should be stressed that we envisage both stages as running continuously and in parallel (Figure 1).

The model that we describe here is limited to a phenomenological proof of concept that instantiates our previously published conceptual ideas [42] in a computational form. Although we are ultimately interested in understanding the neural mechanisms that underpin perception, here we are concerned with providing some insights into the dynamic discovery of the constituents of the competition thought to underlie perceptual organisation, and the nature of the competition necessary to simulate perceptual switching consistent with human experience. The first stage of the model is for now expressed in an algorithmic form, although we aim in due course to implement the functionality identified within a neural architecture. What determines the attractors in our model and how are they discovered? A substantial body of evidence suggests that the brain detects patterns in the incoming auditory signal, which are encoded in sensory memory and subsequently operate in a predictive capacity [13], [43]–[45]. The hypothesis that predictability underlies auditory object representations is discussed at length by Winkler et al [1], [42], and receives particular support from studies of mismatch negativity (MMN) [46] and a number of other electrophysiological indicators that signal the detection of deviations from expected patterns [47], and more recently from neurophysiological experiments as well [48]. Motivated by these ideas, the model we propose acts to discover patterns or regularities in the incoming sequence, and does so by probabilistically creating links between incoming events to construct many potential patterns in parallel. Once discovered, each predictable pattern forms a temporally persistent representation that generates expectations of incoming events. These representations thus represent testable hypotheses about the world and give rise to the dynamic set of attractors in our model. Each representation is strengthened by the rate at which it successfully predicts events and weakened (or eliminated) by erroneous predictions.

How can competition between attractors account for the perceptual experience of human listeners? The predictive representations discovered by the model form a candidate set of perceptual objects, or *proto-objects* [49] that have the potential to emerge as the perceptual objects in conscious awareness. However, all proto-objects cannot emerge at the same time. For example, in the auditory streaming paradigm described above, the galloping rhythm of the ABA−ABA−…pattern is not generally perceived when the segregated organisation, consisting of the A− and B−−− patterns, is reported. What form should the competition take in order to allow only those combinations of proto-objects that are simultaneously perceived to simultaneously emerge as dominant in the competition? This question was addressed by Winkler et al [42], who defined a *compatible* set of proto-objects as a set of predictive representations which together explain the totality of the sensory input without overlap. For any sound sequence there may be more than one compatible set of proto-objects; each set essentially defines a *perceptual organisation* (i.e., one possible interpretation of the sensory scene). Winkler et al argued that such perceptual organisations could emerge from a competition in which attractors competed if, and only if, they predicted the same event; competition was thus proposed to be local both in time and in feature space [42]. As a consequence, we implemented a form of competition amongst the proto-objects that ensures the emergence of compatible proto-objects and gives rise to dynamic switching between compatible sets (perceptual organisations), consistent with human perceptual experience. In this way our model can account for many important characteristics of perceptual multistability observed in auditory streaming experiments, including the typical perceptual organisations reported, the influence of stimulus parameters on perceptual dominance and phase durations, qualitative differences between first and subsequent perceptual phases and the apparent build-up of segregation. In what follows we refer to the proto-objects discovered by the model as “chains” [50], a compact term, descriptive of the way these representations are formed; and we thus refer to the proposed model as the Chains model. (The source code of the model is available as Supporting Information S1.)

## Models

### Events and Chains

The elementary units handled by the Chains model are called *events*. Events correspond to the discrete tokens that comprise a sound sequence. Event onsets elicit a series of electrical brain responses starting with the auditory brainstem response and culminating in the N100 ERP response [51]. For example, the tones that constitute a tone sequence as well as abrupt spectral (frequency or intensity) changes in a continuous sound are considered to be events. It is assumed that the decomposition of a sound signal into events has already been accomplished at an earlier stage of processing, and the task of Chains is to organise sequences of events. This is a simplification, as event detection may also be influenced by the sequences detected in this processing stage (cf. the “Events” section in Discussion).

**Figure 2:**
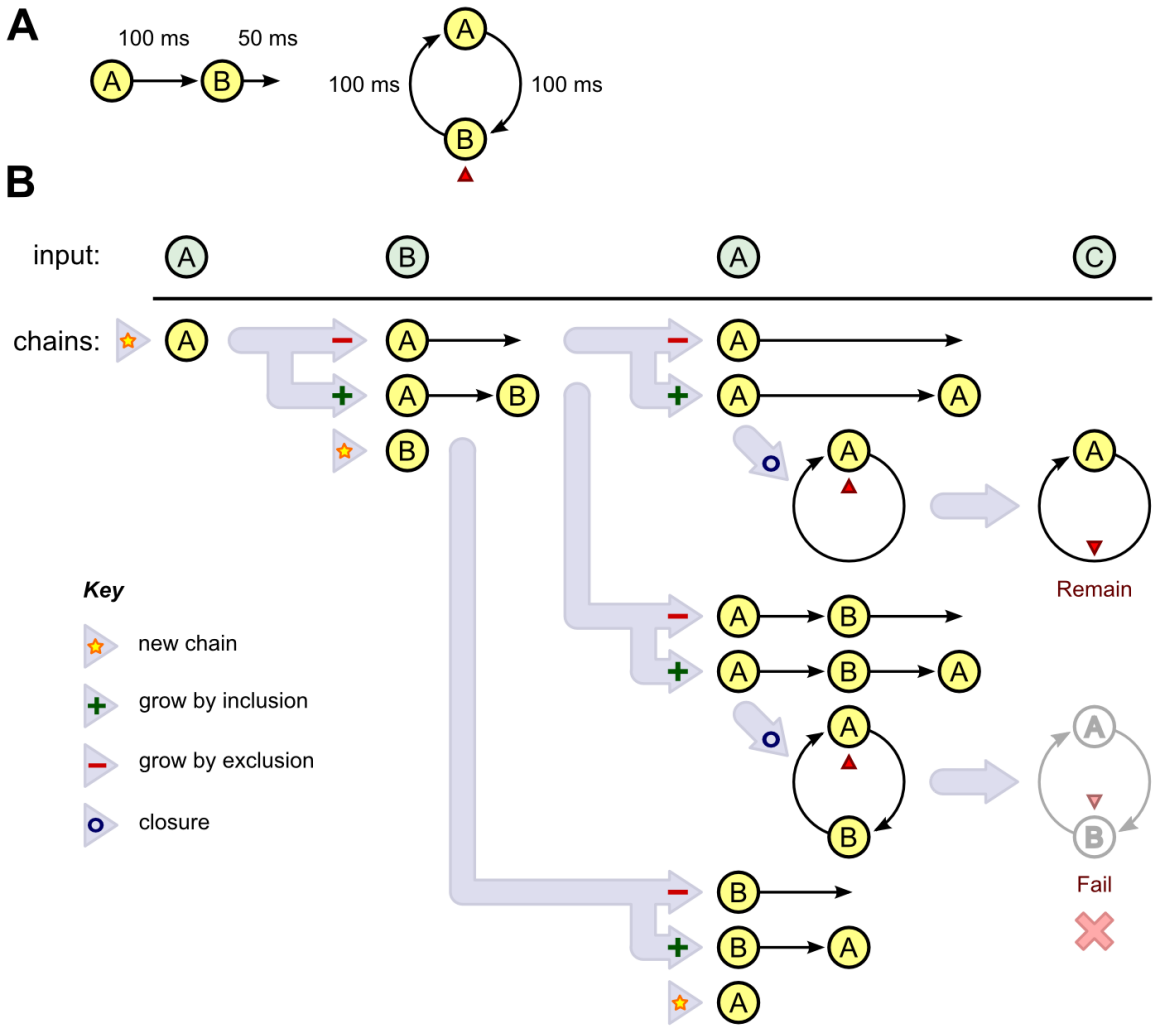
Building chains. A) A linear chain describing event A, followed by 100 ms of silence, event B, which is then followed by 50 ms of silence; and a looping chain which alternates between events A and B every 100 ms. The red “phase” triangle indicates that the looping chain is currently passing through event B. B) The building of chains in response to the input sequence ABAC. Each input event adds a new singleton chain and causes existing chains to split into two new versions (marked by wide arrows), one adding the event and the other omitting it. When A is input for a second time, two potential loops are noticed: AB← and A−←. When event C arrives, the first of these fails to predict B correctly and is therefore removed.

The Chains framework does not prescribe the format that input events should take, nor does it rely on access to the absolute value of any feature of an event. However, it does assume that the *distance* between any given pair of events is available. Inter-event distance measures may be based on a composite of multiple sound features, such as pitch, location and intensity (see Discussion). At this point, evidence regarding the interactions between features in auditory streaming is insufficient for a thorough consideration of the issue. Therefore, the concrete examples given here rely on well-documented effects of frequency differences. We assume that the events are pure tones differing only in their frequency, and that the distance, 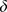, between two tones with frequencies 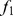 and 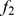 (Hz) is specified in semitones, i.e.,
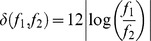



As input events arrive, they are incorporated dynamically into graph-like structures called *chains*, which describe a temporal sequence of sound events. We describe two types of chain: an *open* chain, that absorbs and grows with input events, and a *closed* chain, that does not grow but provides testable predictions of input events. In this work, an open chain consists of a linear sequence of events, and a closed chain consists of a loop—although many other open and closed chain types are undoubtedly conceivable (see Discussion). Open and closed chains are depicted diagrammatically as graphs whose nodes and arcs correspond to events and time intervals, respectively (Figure 2A). Under isochronous sound presentation, chains are notated textually using letters for events, dashes (−) for silent intervals and ← for closure.

### Discovering perceptual patterns: Chain assembly

The basic life-cycle of a chain can be sketched in terms of four stages, depicted in Figure 2B. Firstly, the arrival of an input event triggers the formation of a new open chain, which is then maintained in parallel alongside any other existing chains. Secondly, a chain grows as time passes, and incoming events may be either included or excluded (see “Forming links” for the rules of inclusion and exclusion). Thirdly, if a chain shows evidence of repetition, it closes to form a loop, which signifies the discovery of a possibly repeating, hence predictable, sequence (see “Chain closure”). The closed chain subsequently *predicts* events according to the pattern it encodes, *suppresses* predicted input events (making new chains using those predicted events harder to build) and *competes* with other chains for dominance (see “Competition for perceptual dominance: Chain dynamics”). Fourthly, chains cannot grow indefinitely: an open chain is deleted if it grows too long without closure, and a closed chain is deleted if it makes incorrect predictions. The deletion of chains is a simplification included in the current version of the model; MMN studies showing diminishing MMN amplitudes elicited by successive deviant events [52], [53] suggest that incorrect predictions cause regularity representations to be weakened rather than eliminated, and furthermore, even regularity representation violations which no longer elicit the MMN can be reactivated by a “reminder” event [54] (cf. Discussion).

### Starting chains

As mentioned above, each time an input event arrives, a new open chain is created consisting solely of that event. These singleton chains grow as links with later events are established.

### Forming links

With each incoming event, each open chain has the potential to form two new chains, either by incorporating the event (*inclusion*), or by leaving it out (*skipping*). This include-or-skip principle enables predictable sub-sequences embedded within a more complex sequence to be discovered; i.e., open chains can build representations that skip over events, thus potentially finding repeating patterns that do not include all sounds within the sequence.

**Figure 3:**
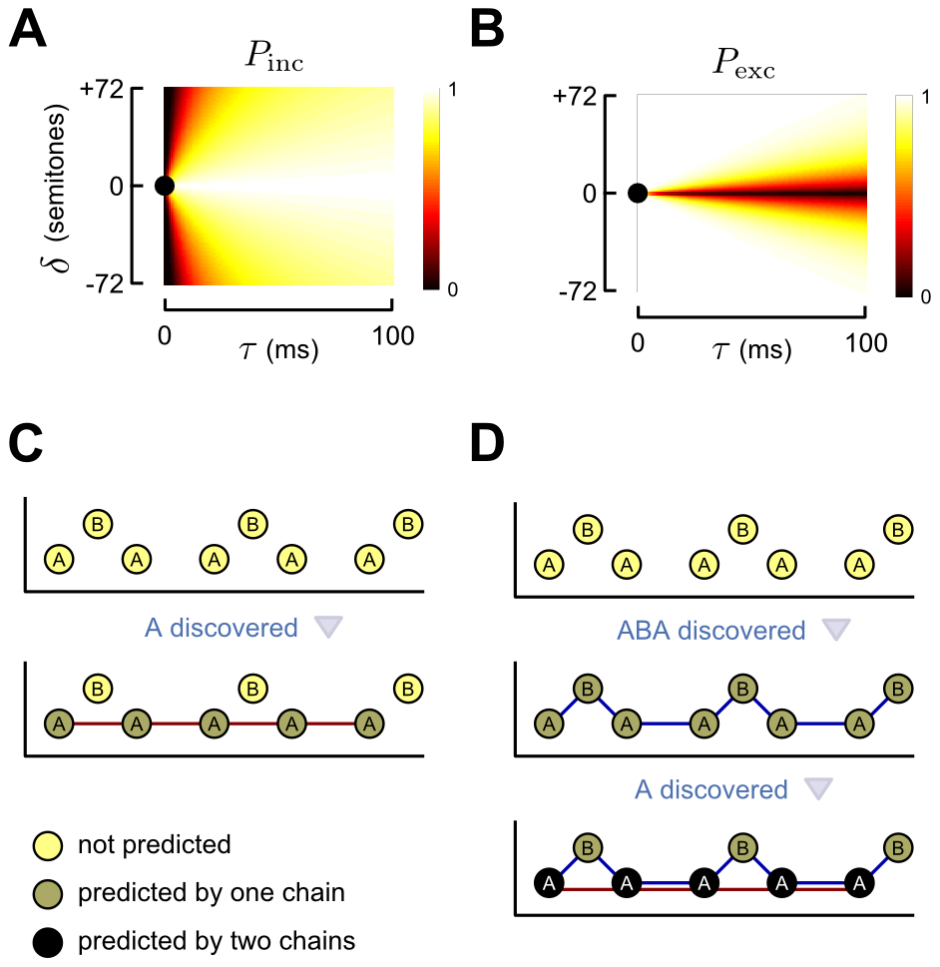
Forming links. A) Profile showing the probability that an event is added, given the parameters listed in Table 1 and assuming 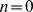. The preceding event is denoted using a heavy black dot, the event to be potentially added is characterised by its temporal distance, 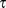, and feature distance, 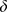, from the previous event. B) Profile showing the probability that an event is omitted, given the parameters listed in Table 1 and assuming 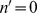. C) The role of 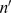 when segregation is discovered first. Top: the input sequence. Below: Once the A−← chain is discovered, A is predicted by one chain and B is not predicted, so 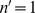 in the exclusion formula for excluding A from a chain currently ended by B. This difference thus facilitates the construction of −B−−←, because the A events are easy to exclude. D) The role of 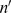 when integration is discovered first. Top: the input sequence. Middle: When the ABA−← chain is discovered, both A and B are predicted by one chain, so 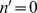. Bottom: However, once A−← is also discovered, A is predicted by two chains and B is predicted by one chain. Thus, 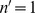, again facilitating the construction of −B−−←.

*Inclusion*. We first discuss the probability of including an event. Let 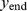 denote the last event in an open chain and 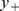 denote the event to be potentially added. Let 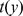 denote the time of event 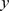 and 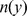 denote the number of competing chains that predict event 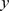. (Note that by predicting event 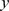 we refer to a specific event, including its features as well as its timing). The probability that an event is included is then given by
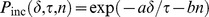
where 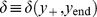, 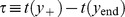, 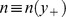. Inspecting each component of the exponential in turn, we see that a connection is *less likely* to form, if (i) the transition from one event to the next is abrupt (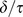 is large, events are dissimilar); or (ii) the input event is predicted by many other chains (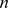 is large, ‘explaining away’ [55]). The relative contributions of these two factors are controlled by the parameters 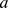 and 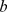, respectively. The probability that an event is included in a chain is depicted in Figure 3A as a function of 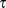 and 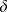 (assuming 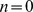).

*Skip*. Similarly, continuing a chain by skipping over an event is associated with a probability,
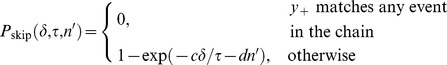
where 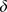 and 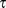 are defined as above, and 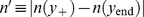. Thus, examining the second expression, we see that a chain that skips over event 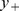 is *more likely* to be built, if (i) the transition from one event to the next is abrupt (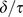 is large, events are dissimilar); or (ii) the 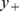 and 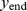 events differ greatly in the extent to which they are predicted by other chains (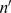 is large). For as long as two events are predicted by a different number of chains (i.e., 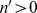), there is a contrast between them which provides evidence in favour of their segregation and thus favours the exclusion of 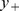 from the chain containing 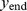 (i.e., suppression). In this way the existence of other chains can support the exclusion of an event from a given chain; e.g. see [56]. This principle is explained diagrammatically in Figures 3C and 3D. The relative contributions of the similarity and suppression factors are controlled by the parameters 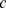 and 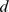, respectively. The first expression (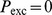) means that if a chain incorporates a particular event, it cannot skip over the same event later. This too is a simplification, and essentially encapsulates the classical notion of exclusive choice between integration and segregation (cf. Discussion for further consideration of this issue). The probability that an event is omitted from a chain is depicted in Figure 3B as a function of 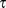 and 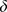 (assuming 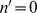).

In summary, for each input event, any given open chain may split into two new chains, one which includes the new event, and one which skips over it but remains able to continue building. A brief glance at the expressions for 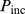 and 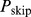 (or their graphical counterparts in Figure 2) reveal that, in general, the easier an event is to include, the harder it is to skip, and *vice versa*. Since they are probabilistic, it is also possible for inclusion and skip to both fail, in which case the open chain in question is simply deleted; i.e., in neural terms any activity associated with this sequence is assumed to be extinguished.

### Matching events

In the course of building chains there is the need to make discrete decisions as to whether two events are the same on the basis of continuous measures of differences between them (e.g., differences in their frequency or timing). Clearly, demanding that the continuous variables match exactly is ruled out by the physiological imprecision with which events are encoded, so instead we introduce thresholds to serve as decision rules. Specifically, two events 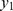 and 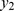 are judged to match if
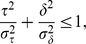



Where 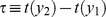, 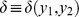, and 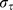 and 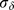 are parameters that specify the dimensions of an elliptical matching region in a time-feature space. Consequently, decreasing either 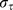 or 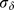 results in a stricter matching criterion. If a decision is to be made as to whether two events occur at the same time (regardless of their similarity), or are of the same type (regardless of their timing), then matching is made on the basis of the inequalities
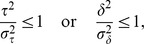
respectively. Clearly, this is another simplification, as similarity/matching is context-dependent (e,g., [57]) and thus it may be affected, for example, by the currently dominant perceptual organization.

### Chain closure

Open chains, which are formed by stringing together input events, must eventually be discarded or converted to closed chains, which are immutable and predict input events. This procedure is called *closure* and occurs when, following the addition of an event to a chain, the first and last events match. When obtaining the closed version of an open chain, it is also converted to a loop by returning the arc exiting the second-to-last event to the first event in the chain. (The requirement for discovering periodically repeating patterns is another relaxable simplification of the current model, which we consider further in the Discussion.) A copy of the original open chain is also retained in the chain population. Closure is illustrated in Figure 2B for the input sequence ABAC. At the point where the open chains A−A and ABA arise, two closed chains are constructed: A−← and AB←.

Closed loop chains continue to cycle periodically through the list of events they contain, making predictions according to the temporal pattern that was established during the chain building process. For each prediction that a closed chain makes, there must be a matching event in the input sequence. If there is not, then the chain fails and is removed. (For further consideration of this simplification, see Discussion.) However, the converse does not apply: a closed chain need not predict every input event. Thus, referring to the example in Figure 2B, the AB← chain fails when the input event C arrives, because the predicted B event is not matched; but the A–← chain persists, because it makes no event prediction at that time.

The parameters of chain assembly are summarized in Table 1, along with sample parameter values.

**Table 1:** Chain building parameters.

Name	Description	Value
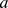	effect of rate-of-change on 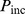	0.00015
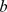	effect of number of times an event was predicted on 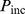	1
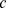	effect of rate-of-change on 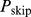	0.0055
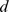	effect of difference in number of times two events were predicted on 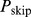	8
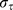	temporal matching width	30 ms
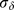	event distance matching width	0.5 semitones
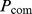	probability of chain first entering competition	0.2

Parameters that control the construction of chains and the matching of events. (See the “Discovering Perceptual Patterns: Chain assembly” section in Models for details.).

### Chain competition

Once a closed chain completes a single cycle of (correct) predictions, it either becomes *competitive*, with probability 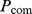, or it is deleted, with probability 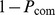. Prior to becoming competitive, all incoming events which the chain contains must be successfully predicted, and all incoming events which the chain does not predict must be successfully skipped. Upon becoming competitive, a chain acquires a *dynamical state*, which allows it to interact with other chains.

A parallel to this two-stage processing of closed chains can be found in deviance detection experiments. Some studies have shown that the deviance detection process reflected by the MMN event related potential (ERP) response is only elicited by a deviant sound arriving after at least two repetitions (three presentations) of the regular sound [53], [58], [59]. One could argue that the first repetition forms a possible predictive regularity (“closing the chain”), whereas the second repetition activates it (i.e., enters it into the competition). However, when the sounds are attended, deviants encountered after a single repetition elicit the MMN [60]. Thus by means of attention one may vouchsafe a regularity representation and so skip the “sanity-check” cycle.

### Competition for perceptual dominance: Chain dynamics

**Figure 4:**
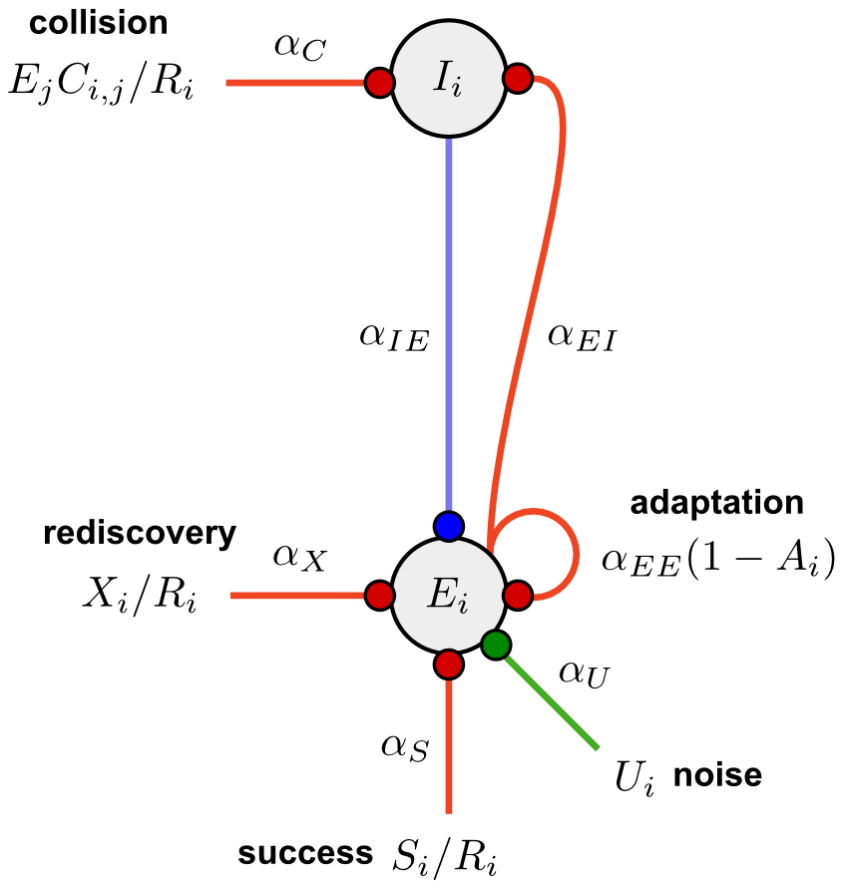
Dynamics associated with a single chain. Each chain, 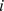, is associated with an excitatory and inhibitory population of abstract neurons (discs). The termination of each arc onto a population denotes an additive term, which affects the activity in a population (see equations 1 and 2). A red terminal indicates that the influence is always positive. A blue terminal indicates that the influence is always negative. A green terminal may be positive or negative in its influence. The source of each arc in the diagram denotes an additive term in equation (1) or (2), the expression attached to each arc is the coefficient which scales that effect. Note that this diagram shows the dynamics associated with chain 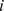, and all other chains are referred to as 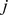.

Having described how chains are initially built, make predictions, and become competitive, we now discuss how the competition is mediated. Each competing chain (hereafter, simply “chain”) is represented by a population of excitatory and inhibitory neurons [61], [62], associated with eight state variables: 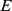, 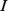, 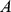, 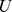, 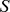, 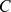, 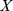 and 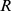 (see Table 2 for a short description). Throughout the text, we will indicate the chain to which a state variable belongs by a subscript 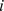. The way in which the dynamics of a single chain is modelled using the two hypothetical populations of neurons is illustrated in Figure 4. The 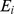 and 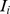 state variables represent the activation levels of the excitatory and inhibitory neuronal populations of chain 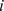, with 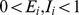, and their dynamics are governed by the following equations:
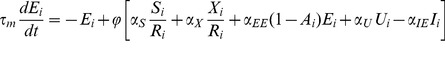
(1)

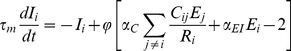
(2)where 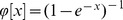 is the standard sigmoid function, which constrains the range of excitation (and inhibition) to the target range 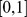 and ensures smooth asymptotic, rather than abrupt, convergence to these limits. Note that 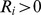 (see below), preventing divisions by zero in both equations. Equations (1) and (2) calculate the amounts by which the levels of excitation and inhibition associated with each chain 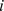 (see Figure 3) change during the characteristic time period, 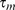, as a result of the previous excitatory/inhibitory state and the additive effects modelled (including interaction with other chains). Thus (1) and (2) take the form of differential equations, relating the rate of change of excitation and inhibition to the state variables (modelled effects).

**Table 2:** Dynamical state variables.

Name	Description
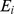	excitation
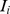	inhibition
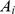	adaptation
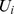	noise signal
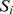	success rate
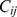	collision rate with chain 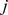
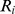	input event rate
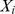	rediscovery rate

Eight continuous variables (functions of time) that describe the dynamical state of chain 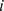 at any given moment. Note that all variables except 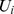 are always non-negative, and 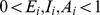. All dynamical variables are initialised to zero upon a chain entering the competition. (See the “Chain Dynamics” section in Models for details.).

The level of excitation for each chain (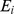) determines dominance among chains and thus which percept appears in perception. 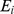 is the ‘output’ term for each chain and the only globally accessible quantity; all other terms and variables are internal and private to the model for each chain. The level of inhibition for each chain (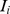) mediates the competition between them. By passing all the inhibitory contributions to the excitatory population through a local inhibitory population we prevent excessive inhibition and ensure that the level of inhibition is constrained to lie between 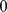 and 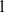; the 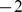 term is necessary to avoid saturation. The various effects governing the dynamic state of each chain are modelled as additive processes, mediated by the sigmoid function. The role of each of the variables, their intuitive meaning, and associated parameters are described and motivated in the paragraphs that follow. The 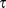 (time constant) and 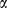 (effect magnitude) parameters which appear in (1) and (2) are listed in Table 3.

**Table 3:** Dynamical system parameters.

Name	Description	Value
	*Time constants*	
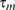	excitation and inhibition	50 ms
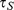	success rate	1 s
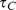	collision rate	1 s
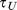	noise fluctuation	500 ms
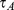	adaptation	5 s
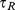	input event rate	5 s
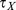	chain rediscovery rate	5 s
	*Coefficients*	
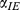	inhibition to excitation	8.1
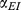	excitation to inhibition	1
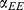	self-excitation	3.2
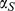	success rate	3.8
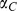	collision rate	3
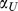	noise	3.4
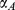	adaptation	0.1
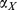	chain rediscovery	7
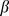	denominator term	0.1

Parameters that control the dynamics of competing chains. The 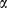 parameters control the magnitude of the corresponding effect, and the corresponding 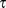 parameters are time constants associated with the same effects. (See the “Chain Dynamics” section in Models for details.).

**Figure 5:**
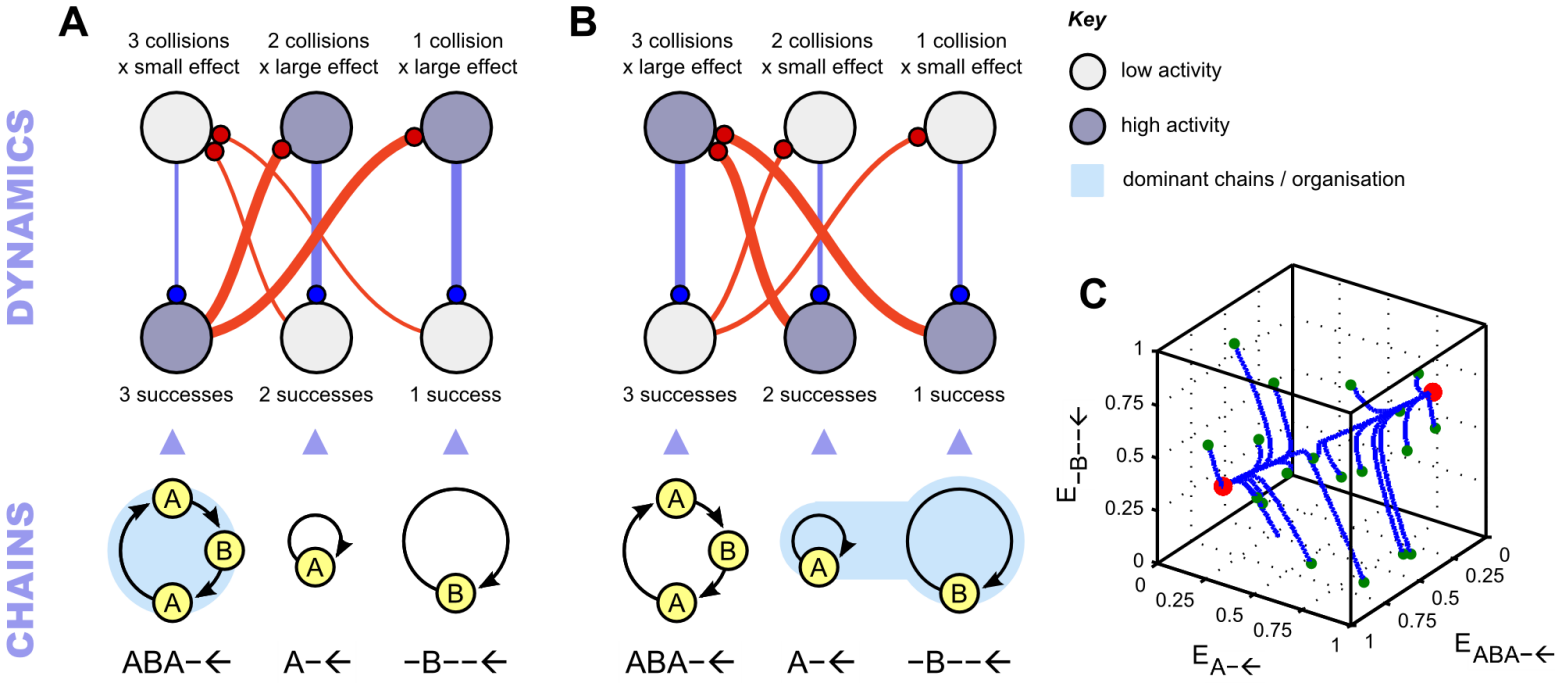
Dynamical system formed in response to an ABA− sequence. **A and B**) Collision and success rate effects shown on the excitation/inhibition (dynamics, top; see Figure 4) and the sound-group depiction (chains, bottom; see Figure 2) of the chains formed in response to a repeating ABA− sequence. Columns represent the three most stable chains formed: ABA−←, A−←, and −B−−←, from left to right. DYNAMICS (top panels): The inhibitory neuronal population is shown at the top, the excitatory one at the bottom of the panel. The strength of each population is marked by the filling of the circles (empty circle = weak, filled circle = strong). The size of the suppressing effect of the inhibitory population on the excitatory one is marked by the width of the blue line connecting them. The inhibitory population of a chain is strengthened by collisions with other chains (see section “Successes (*S*) and Collisions (*C*)”); the number of collisions and the amount of strengthening they provide to the inhibitory population of the given chain are noted over the inhibitory population. The effects of collisions are marked by red arcs connecting the excitatory population of each chain with the inhibitory population of those chains with which it collides (A−← and −B−−← don't collide, all other pairs do). The size of the strengthening effect to the inhibitory neuronal population (dependent on the strength of the excitatory population of the other colliding chain) is marked by the width of the arc. Excitation is strengthened by the rate of successful predictions made by the given chain; the number of successful predictions is noted below the excitatory population. For simplicity, the rediscovery, noise and self-excitation terms are not depicted here. Chains (bottom panels): Blue shading marks the currently dominant chain (i.e., the chain(s) whose excitatory population is stronger than that of the other chains). A) *Integrated organisation dominant*. Whilst the ABA−← chain dominates, the excitatory activity associated with the A−← and −B−−← chains is low. B) *Segregated organisation dominant*. Whilst the A−← and −B−−← chains dominate, the excitatory activity associated with the ABA−← chain is low. The events in the A−← and −B−−← chains do not collide with each other, so they have no inhibitory effect on each other. C) System state showing the various trajectories that the 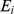 variables associated with the three chains (represented by and marked on the three axes) take, given 20 randomly-chosen initial values (green dots). In the absence of noise, the system permanently settles into one of the two organisations associated with diagrams in (A) and (B) (red dots), moving along a deterministic trajectory (blue lines). That is, some time after the start of the sequence either ABA−← becomes highly excited with A−← and −B−−← becoming weak (lower left red dot) or *vice versa* (upper right red dot) and the excitation and inhibition values of the three chains do not change anymore (i.e., the model without a noise effect would predict stable perception).

For a suitably chosen set of parameters (see Tables 1 and 3), the interplay of the modelled effects defines an attractor landscape whose stable states correspond to the perceptual experience of listeners. The excitation variables of the chains tend to assume either relatively “low” or “high” values (corresponding to the attractor states), with intermediate ones only very fleetingly present. We refer to the highly-excited chains as *dominant*. Consider the interactions between the three chains that most often form in response to an ABA− sequence; i.e. ABA−←, A−← and −B−−←. In this case, two stable states are possible. In the first, the ABA−← chain is dominant (i.e., it is in a highly excited state) and the A−← and −B−−← chains are non-dominant (i.e., they are in low-excitation state); in the second, the A−← and −B−−← chains are both dominant, and the ABA−← chain is non-dominant. These two network states are illustrated in Figures 5A and 5B, respectively. We refer to these stable configurations of the excitation states of chains as *organisations*, by analogy with the perceptual organisations that spontaneously emerge when a listener is presented with an ambiguous stimulus.

### Successes (*S*) and collisions (*C*)

The competition between chains depends primarily upon successes and collisions in their predictions. A *success* occurs whenever a chain correctly predicts an event. Correctness of a prediction is decided by checking whether an input event occurs within the matching region of the prediction (i.e., it is close to the prediction both in time and in its feature values; see section “Matching events”). A *collision* occurs between two chains whenever they both predict the same event: a prediction of one chain is within the matching region of a prediction by the other chain. If predictions are separated by either temporal or featural differences, or both, then they do not collide. As was mentioned before, during competition, chains interact with each other only when they predict the same incoming event (i.e., they collide with each other). Successes and collisions of chains form separate point processes, assessed by the state variables 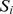 and 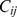, respectively, which are essentially running averages of these occurrences maintained by leaky integration, i.e.,
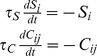
and
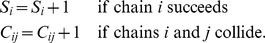



Notice that 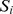 and 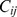 are expressed in units of successes or collisions per second, respectively, and they are incremented when the corresponding event occurs.

Returning to equations (1) and (2) above: the term 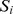 contributes positively to the excitatory variable 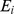, and the term 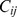 contributes positively to the inhibitory variable 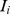. Consequently, chains are excited to the extent that they succeed in their predictions, and are inhibited to the extent that they collide with other excited chains. 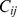 appears in the product 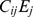, so the effectiveness of the collision upon chain 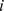 is modulated by the level of excitation of chain 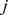. That is, the size of the inhibitory effect of one chain on another chain with which it collides is proportional to its current level of excitation, weighted by the coefficient 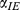. The coefficients 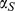 in equation (1) and 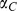 in equation (2) are the parameters which control the magnitudes of the success and collision effects on excitation and inhibition, respectively; 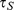 and 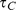 are the time constants that determine how long these effects last (see Table 3). As described above, the inhibition associated with each chain is private to that chain, and simply keeps track of the rate of collisions with other chains. It is also driven by its associated excitatory population, mediated by the coefficient 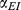.

### Chain rediscovery (*X*)

Since the chain building process is assumed to be ongoing, any chain that has entered the dynamical competition may be discovered again later. Rather than allowing two (or more) equivalent chains into the competition, a chain 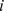 is formed just once, and if duplicates arise later, the variable 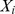 is incremented, and the copy is discarded. This variable is governed by
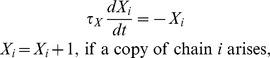
and it constitutes a moving average of the rate at which a particular chain is rediscovered. This term ensures that the competitiveness of each grouping is related to their likelihood of discovery and thus provides an estimate of the “strength” of the grouping represented by the chain; i.e. those patterns that are easiest to discover tend to dominate the competition. The coefficient 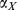 in equation (1) is a parameter which controls the extent to which a chain being rediscovered supports its dominance in the competition (excitation value); 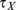 is a time constant that determines how long this effect lasts.

Comparing two chains requires matching the events that they contain to ensure that the first chain does not contain any event that the second chain does not, and *vice versa*. Special attention is required when comparing looping chains, as the events they contain may match initially but drift out of phase over a long time period. Here for simplicity we compare two chains over a time/event “horizon” of two cycles; that is, two chains match if they predict matching events over the course of two cycles.

### Normalisation (*R*)

It is desirable that the model remain well-behaved when the same sequence is presented at different presentation rates. The divisive factor 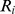, which is attached to the success, rediscovery and collision terms in equations (1) and (2), serves the purpose of normalising the switching of the model when events are presented in the same pattern but at different rates (similar to the rate normalisation applied in [63]).

The variable 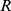 is computed by integrating the overall rate at which input events arrive, according to the following equation
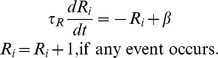



The normalisation variable is averaged over a longer time scale than the success and collision variables (i.e., 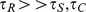; see Table 3). This means that the model can be tuned for the ratios 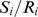 and 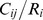, which are invariant with respect to rate for a given input pattern, rather than 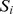 and 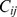. The constant 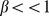 is included purely to prevent division by zero.

Note that this normalization does not eliminate all effects of presentation rate. Presentation rate determines the rate at which the system has opportunities to form links, and thus there is inevitably a slower discovery of chains with slower presentation rates. In the Results section we provide empirical evidence, captured by the proposed model, suggesting that, whereas the choice of the initial percept and its duration is governed by variables (including presentation rate) affecting the discovery of the alternative proto-objects (chains), competition between the already discovered alternatives is far less sensitive to these variables and thus to presentation rate. However, some effect of the link-formation variables (amongst them presentation rate) on the ongoing competition is detectable in perceptual behaviour and this is modelled through the rediscovery rate (represented in the model by 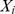).

### Noise (*U*)

For any given initial condition, the dynamics of the model as described thus far will eventually settle into one of the organisations. This is demonstrated for the ABA− example in Figure 5C. In order for Chains to exhibit multistable switching, a source of noise is required to destabilise the system to a degree which suffices to “jolt” the system's state from one attractor to the other. This source of noise is provided by the state variable, 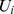, which evolves according to the state equation 
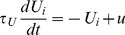
where 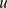 is a Gaussian random variable with zero mean and unit variance. The noise signal 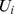 belongs to the class of Ornstein-Uhlenbeck random processes [64] and appears as an additive term in the net input to the excitatory activity 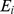. These slowly fluctuating noise processes have been previously employed in perceptual bistability models [20]. All noise signals are decorrelated and there is no direct noise input to the inhibitory populations. The corresponding coefficient in equation (1), 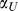, regulates the relative impact of the noise and hence how often the model switches; the larger 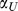 is, the faster the switching rate. The time constant 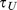 controls the fluctuations in the noise signal; large values for 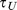 (i.e., slower time constants) preclude fast fluctuations.

### Self-excitation and adaptation (*A*)

In addition to the basic switching behaviour established as described above, we also introduce a *self-excitation* term, which prolongs the time spent in a given attractor by reinforcing the current state. Self-excitation is proportional to the current excitation level. Cross-inhibition and self-excitation can lead to the dominant percept remaining stable indefinitely within certain parameter regimes [19]. Hence, models of bistability generally include some form of adaptation. Adaptation is a well-known phenomenon both in behavioural and neural studies of sensory processes. Repeated exposure to the same input reduces the response to this input (see, e.g., stimulus-specific adaptation [65], [66]). In a similar vein, several mechanisms are capable of bringing about adaptation within a neuronal population, including neuronal fatigue (typically modelled by firing rate adaptation; cf. adaptation-LC model in [67]), self-excitation with synaptic depression ([68] and the present work), increasing levels of recurrent inhibition, or adapting inhibition from competing populations. These various forms of adaptation and their impact upon bistability are explored in depth in [19], [67]. It is not uncommon for models to incorporate multiple sources of adaptation (e.g., [68]). Our model incorporates two: an adapting self-excitation, and a form of adaptation mediated via the inhibitory population and controlled by the parameter 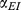. Adapting self-excitation is a sufficient but not a necessary condition for producing gamma-like phase distributions [69].

The self-excitation and its adaptation both depend on the level of current excitation, although they have the opposite effect on the excitation level of the chain. The *adaptation* state variable, 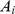, modulates the efficacy of the self-excitation and evolves according to the equation
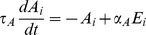



**Figure 6:**
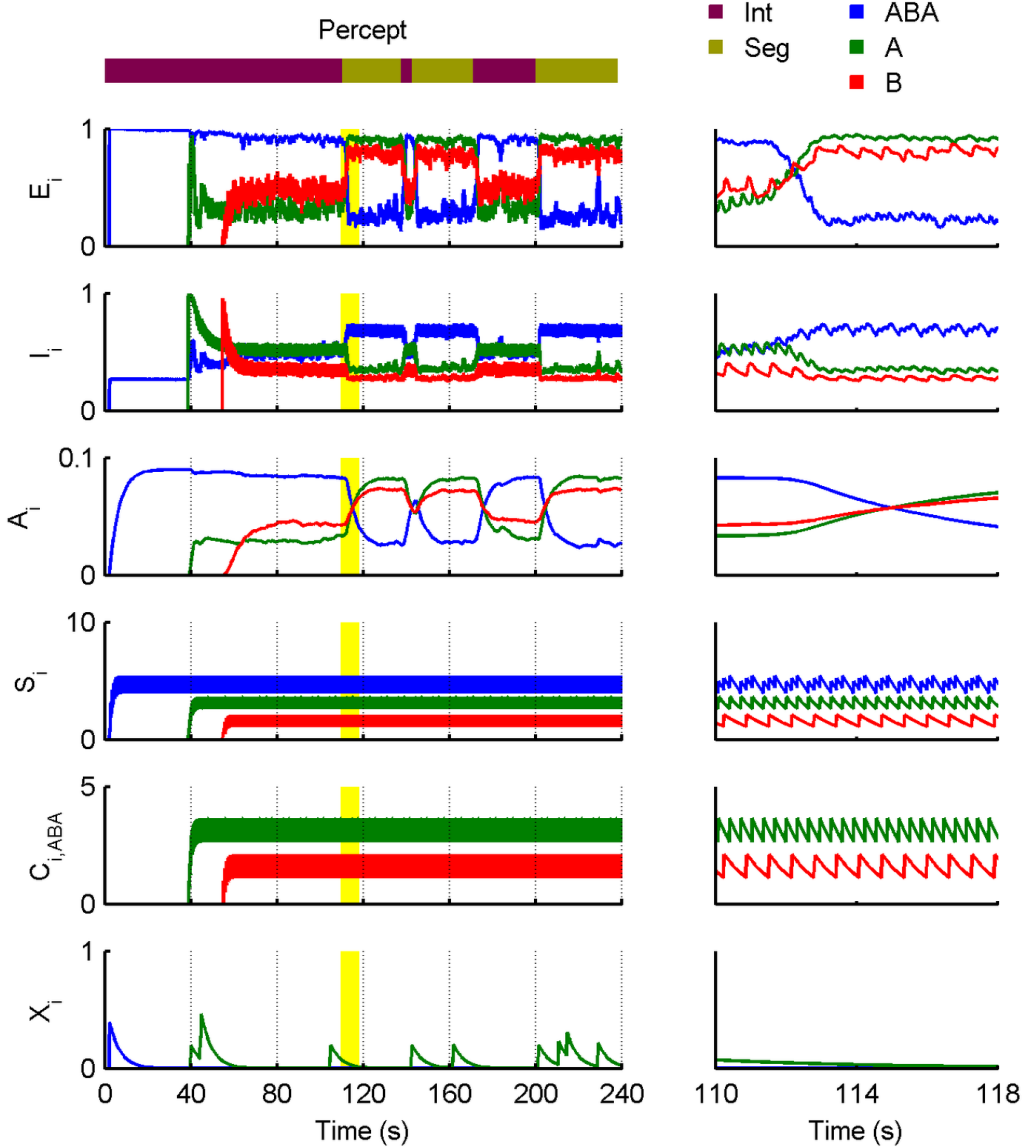
Dynamical switching. The *left panels* show the excitation and other dynamical state variables of the chains that arise in response to a four-minute long ABA− sequence with 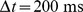, 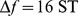. The excitation variables (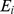) alternate at random intervals between two stable organisations once they are both discovered (at around 40 seconds): “integrated” (blue only) and “segregated” (red [“B”] and green [“A”] together). The percepts that would correspond to the chain with maximum momentary excitation are plotted above, calculated from low-pass filtered excitation time-courses (to avoid bouncing). Segregation dominates 74% of the time; the mean phase duration is 23.7 s. The *right panels* plot the changes in the state variables during a perceptual switch at 110 seconds on a magnified time-scale. The corresponding time period in the left panels is highlighted in bright yellow. Chain excitations are modulated by the noise variables 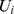 (not shown). The inhibitory populations (with activities 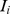) serve to achieve exclusivity of the stable organisations by suppressing chains colliding with the dominant one. The adaptation and self-excitation state variable (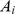) renders switches in close succession unlikely (self-excitation) while increasing the probability of a switch as the duration of the perceptual phase grows (adaptation). The probabilistic rediscovery of a chain supports its excitation through the rediscovery rate (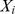).

Consider the state of the system immediately following a switch into the attractor associated with excitation for ABA−← in the three-chain description of the ABA− sequence as shown in Figure 6. Initially, this chain is little adapted as the level of adaptation declines when the chain is in a low excitation state, so upon switching to the high excitation state, self-excitation is initially high; this drives the excitation level of the chain up and makes it unlikely that a switch to the opposite organisation will occur. Whilst 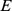 remains high, the chain adapts and thus self-excitation subsides; at the same time, due to the low 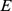 values associated with the A−← and −B−−← chains, they recover from adaptation. When a switch eventually occurs, the same self-excitation/adaptation cycle occurs again. The panels in the right-hand column of Figure 6 illustrate how the dynamical variables change during a switch (in this case, from integrated to segregated).

In summary, adaptation coupled with self-excitation encourages the system to remain in the same state in the period immediately following a switch; this reduces the likelihood of very short perceptual phases and produces a distribution of phase durations consistent with a gamma distribution (see Results). The parameters 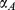 and 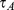 control how rapidly adaptation rises and falls. The level of influence that self-excitation and adaptation has on the excitation of the chain is determined by the coefficient 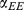 in equation (1).

### Simulating human perception

The output of the Chains model is the set of chains together with their dynamical state at each point in time. This raises the question of how to relate these chains and their states to the responses of listeners in auditory bi- or multistability experiments (e.g., see [41]). Whereas the excitation levels, 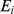, of the ABA−←, A−← and −B−−← chains are continuous and visible to the modeller, the moment-to-moment responses of experimental participants are discrete (i.e., they only record the current organisation reported by the listener), being conveyed via button presses (see the next section). The excitation levels of all other alternative organisations are hidden from the experimenter, although ERP studies may shed some light on the non-dominant sound groupings (see, e.g., [70]). Thus, it is necessary to devise a rule which turns the chain excitations into categorical responses. In the experiment reported here listeners were required to mark their perception as either *integrated* or *segregated*, therefore, we use the following rule for mapping:
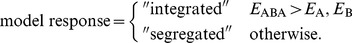



Once this final transformation is included, the model receives a sequence of sound events as input and returns a sequence of perceptual states as output.

### Auditory streaming experiment

The experiment that we use for comparison with the model simulations has not been previously reported, but closely resembles experiment 1 in [41]. All experimental procedures were the same as those reported previously, except for the instructions to the participants, as explained below.

Fifteen healthy young volunteers (9 male, 18–26 years of age, average 21.8 years) with normal hearing participated in the experiment, and received modest financial compensation for their participation. The study was conducted in the sound-attenuated experimental chamber of the Institute for Psychology, Hungarian Academy of Sciences. It was approved by the Ethical Committee of the Institute for Psychology.

The experiment was designed to investigate the distribution of perceptual switching across a relatively large parameter space. Participants were presented with 4-minute long trains of the ABA− structure, where A and B were pure tones of 75 ms duration, including 5 ms linear onset and offset ramps. The frequency of the A tones was kept constant at 400 Hz for all stimulus conditions. In separate trains, the B tones were 4, 10, 16 or 22 semitones (ST) higher in frequency than the A tones. The onset to onset time interval was 100, 150, 200, or 250 milliseconds (ms). Therefore, altogether, 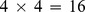 different stimulus combinations were tested (all the stimulus sequences are available in the Supporting Information, Audio S1). The parameters 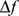 and 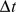 denote the frequency separation and the event onset-to-onset times, respectively.

Participants were instructed to depress one response key so long as they experienced an integrated percept and the other key when they experienced a segregated percept. Thus in this experiment participants essentially had an exclusive choice between integration and segregation and responses with both buttons being simultaneously pressed (occurring less than 2% of the time) were excluded from the analyses. In the experiments reported in [12], [14], [41], [71], [72] this forced choice was relaxed with the result that participants quite often reported other organisations too; we consider this issue further in the Discussion. When participants heard no repeating tone pattern, they were instructed to release both keys. These null responses (appearing less than 4% of the time) were discarded from the analyses. The two percepts (integration and segregation) were explained to participants using auditory and visual illustrations. The experimenter made sure that participants understood the instructions during the training period before the start of the experiment. The state of the two response keys was sampled at 100 ms intervals. Participants were instructed to mark their perception throughout the duration of the stimulus sequence and not to attempt hearing the sound according to one or another perceptual organisation.

The experimental data was analysed as previously reported [41]. Perceptual phase durations (i.e. the time during which a percept was continuously reported), and the corresponding percepts, were extracted. All phases with durations <300 ms were discarded as these were assumed to stem from inaccurate synchronisation of button presses rather than conscious percepts [41], [73]. From this data, we extracted the mean perceptual phase durations and mean proportions of the segregated percept, for each participant and condition. The behaviour of participants during the first perceptual phase was found to be qualitatively different from that during subsequent phases [41], so where indicated we analysed first phase and subsequent phase data separately. Note that in some participant-condition combinations, the number of subsequent perceptual phases were low. These cases introduce some measurement error into mean phase durations. When there were no subsequent phases reported, the missing data were replaced by the participant's average proportion of the segregated percept and average perceptual phase durations across all the conditions in which a subsequent phase was experienced. For the analysis of the temporal dynamics of the percepts we calculated the mean probability of reporting segregation across all participants for each point in time. We then smoothed this data using a moving average with a sliding window of 2 seconds.

In the Results section, in order to compare the model responses with the empirical data, we report simulations from model experiments and take the mean model responses over 15 repeats (cf. 15 subjects; variability in the model responses depends on the noise term) of the 4 minute long stimulus trains for each experimental condition.

We performed the same statistical tests on the simulated and empirical data and compared the significant effects across the two datasets. Specifically, we tested whether the phase durations were drawn from a log-normal distribution by means of Shapiro-Wilk W tests on the logarithms of phase lengths (in milliseconds) in one of the experimental conditions (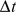 = 200 ms and 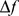 = 16 ST). The effect of 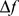 and 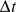 on the choice of the first reported percept were analyzed using Cochran's Q tests, both for all conditions and for all possible pairs of the four conditions corresponding to the corners of the parameter space (short 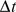 and small 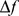, short 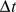 and large 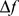, long 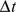 and small 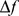, and long 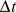 and large 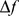). Repeated measures analyses of variance (ANOVAs) were carried out on the first-phase durations and the proportions of segregation and mean durations in the subsequent phases with 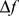 and 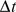 as dependent factors. When applicable, degrees of freedom were adjusted with the Greenhouse-Geisser correction factor (ε). These and the partial η^2^ effect sizes are reported. Post hoc comparisons for significant ANOVA effects were performed using Tukey's HSD tests. All analyses were carried out at the 0.05 alpha level.

### Selection of model parameters

The assembly of chains and their dynamic competition are controlled by the parameter sets listed in Tables 1 and 3, respectively. Although it is technically possible to perform a machine-based optimisation of the free parameters to minimise the distance between the empirical and model data, we chose to fit the parameters empirically in order to gain insight into the influence of each one. Here we review the procedures by means of which the model parameters were determined.

### First phase choice.

The first percept reported corresponds to the first repeating pattern discovered by the model. This is determined by the parameters 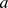 and 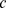, which control the extent to which the rate of change of stimulus features from one event to the next affect the probability of inclusion or exclusion, respectively. The values of parameters 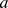 and 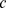 determine whether the influence of temporal proximity (favouring the ABA−← pattern) or similarity (favouring the A−← and −B−−← patterns) predominates. Note that first phase choice is not affected by rediscovery, collisions, adaptation or noise, so the 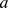 and 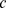 parameters can be chosen without reference to the other parameters (although of course the converse is not true; 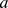 and 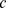 do influence the dynamics of subsequent phases).

**First phase duration**. The duration of the first phase is largely determined by the time taken for *other* chains to be discovered and for them to join the competition. Once the parameters 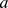 and 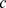 are chosen, first phase duration is controlled by the parameters 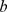 and 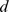 (which determine how sensitive the model is to an imbalance between the number of chains predicting each of the events), and 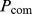 (the probability for a chain that has been formed to enter the competition).

**Subsequent phases: perceptual switching**. Once all chains have been discovered the ongoing competition dynamics is influenced by a number of factors including the rate of successful predictions, collisions, and rediscovery, as well as adaptation and noise. A minimum level of noise, determined by the parameters 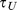 and 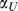, is necessary in order to achieve any switching at all. These parameters were set to ensure that switching occurred, and adjusted later on to fine-tune overall switching rates. The coefficients 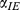, 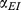, 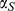, and 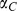 control the excitatory/inhibitory interactions in the populations associated with each chain and the influence of successful predictions and collisions on these interactions. These parameters were adjusted so that competition was balanced for all combinations of 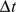 and 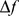, and the mean phase duration was close to the empirical value in the centre of the feature space. Although the influence of the stimulus features on subsequent phase durations is weaker than in the first phase [41], there is nevertheless some effect. This is modelled by means of the chain rediscovery term, controlled by the parameter 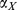; larger 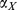 results in larger feature-related differences in dominance durations. Finally, the adapting self-excitation term (controlled by 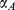 and 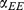) controls the suppression of switching for a period of time after a switch has been made, and the observed approximately gamma-distributed phase durations.

**Time-constants**. The time constants, 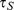, 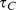, 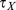 and 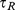, associated with the state variables 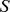, 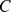, 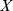 and 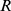, essentially encode the rate of processing in the system; the low-pass filtering controlled by these time constants ensures that the impulses associated with each event enter smoothly into the model dynamics. Consequently, the precise choice of time constants is not important, provided that: (i) they are long enough to encompass a few events in a typical sequence (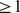 s), otherwise they cannot establish a rate, and (ii) 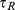 is somewhat longer than 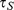 and 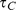, otherwise it cannot serve to normalise the success and collision rates. (Chain rediscovery tends to occur less frequently than successes and collisions, so the time constant 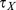 must be somewhat longer in order to provide a running average.)

It should be noted that the perceptual switching behaviour of participants in bistability experiments, both in vision and in audition, varies widely (e.g., see [14], [74]); there may be an idiosyncratic bias towards one or other organisation, and typical switching rates can be very different. The model we propose, with the parameters shown in Tables 1 and 3, qualitatively captures the mean behaviour of human listeners in the streaming experiment reported. While a more precise match may be obtained, we were concerned primarily with exploring the insights the model provides into the principles underlying perceptual organisation, rather than precisely matching a specific data set. However, the parameters used are reasonable and, where possible, constrained by biological plausibility. The same set of parameters was used for all the results reported.

## Results

### Switching organisations

Figure 6 provides an example of the alternation between perceptual organisations that emerges from the competition amongst chains in the model. The simulated data shown correspond to a “neutral” stimulus condition that does not strongly promote either integration or segregation (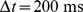,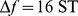). Consequently, all three chains are discovered within the first minute and proceed to compete. The competition yields two stable perceptual organisations, one in which the ABA−← chain dominates (integrated), the other in which the A−← and −B−−← chains dominate (segregated). Perceptual switches occur at random intervals (see Figure 6 caption for statistics).

Figure 6 also shows how the activity of the excitatory and inhibitory populations of the three chains evolves over time, as well as the time-course of self-excitation and adaptation, success rate, collision rate, and re-discovery rate. Note that the excitatory and inhibitory variables associated with each chain remain positive between events. This is due to the fact that dynamical variables (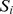, 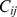, 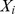, etc.) are filtered with time constants on the same order of magnitude as the inter-event intervals for typical presentation rates (that is, from 100 ms to 1 s). Furthermore, with the exception of self-excitation, the direct contributions to a chain's dynamics (via successes and collisions, etc.) do not dependent on 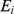, that is, whether the chain is dominant or suppressed. Consequently, even suppressed chains show a degree of excitation.

The left-hand column of panels in Figure 6 show how the variables evolve throughout a full four-minute simulation. However, when viewed on a fine time scale, as in the right column, oscillations in some of the state variables are apparent. For example, success rates (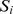) are leaky integrators that oscillate around the number of successful predictions in unit time. The “A” chain predicts an A tone every 400 ms (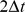). Every time an input event falls within the matching region of the “A” chain's prediction, a success is registered, and 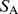 is increased by one. Note that in this model auditory events correspond to tone onsets and predictions are evaluated at these moments. As a result of implementing 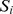 as leaky integrators, the average of success rates over at least 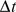 time reflect running averages of successful predictions per unit time. In our example, the “A” chain makes four correct predictions per second (two in an ABA− cycle), the “B” chain two, and the “ABA” chain six as it predicts all the input tones. Similarly, the collision rate variables are leaky integrators oscillating around the number of collisions per second for each pairs of chains. That is, 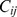 is increased by one every time chains 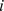 and 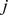 predict the same event. Because the equations governing the excitatory and inhibitory state variables include 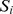 and 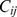 as additive terms, the former inherit the oscillations present in the latter to some extent.

The magnified time period plotted in the right-hand column of panels in Figure 6 highlights not only the presence of fine structure in the state variables, but also their dynamics during a switch. As the excitation (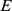) of the “ABA” chain (blue) falls, its adaptation (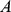) begins to decline, albeit with a longer time constant. Conversely, as the excitation of each segregated chain (“A”, green; “B”, red) rises, the degree to which it is adapted also increases. The rate that a chain adapts depends on its excitation. For example, the “B” chain adapts more gradually than the “A” chain, because it is less excited on average (owing in turn to fewer successes per second). The slower build-up of adaptation for the “B” chain relative to the “A” chain is particularly apparent when each one first appears. (See the four-minute graph of 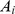 in Figure 6, left column.)

**Figure 7:**
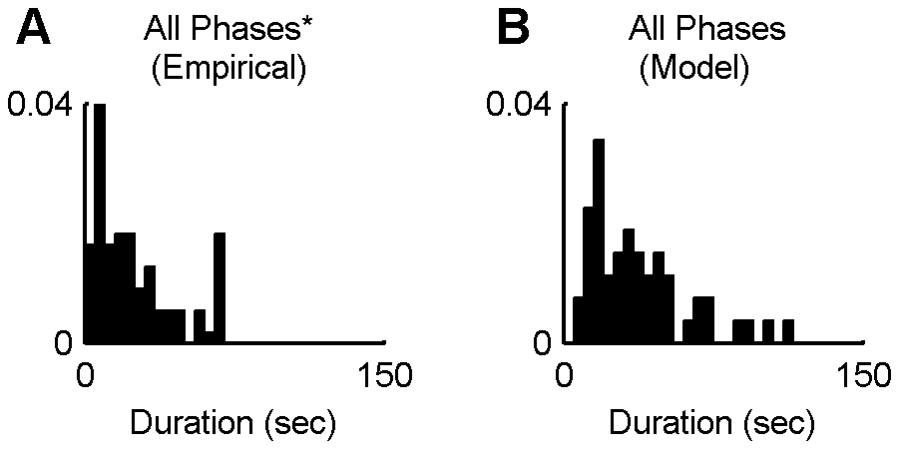
Phase length distributions. A) Distribution of the perceptual phase durations obtained from the perceptual experiment data with 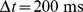 and 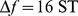 (110 phases from 15 participants). B) Distribution of the “perceptual” phase durations obtained from the model for the same 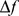 and 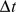 parameters as in panel A) (53 phases from 15 simulations). Note that a small number of outliers are not visible (a 213 and a 223 seconds-long perceptual phase on panel A and a single 179 seconds-long phase on panel B). ^*^Empirical phases exclude “both” and “neither” responses.

The distribution of the phase durations, plotted in Figures 7A and 7B for the experimental and model data, respectively, reveal the gamma-like or log-normal distribution of phase durations generally reported for bistability experiments. However, it should be noted that our experimental data only approximates this distribution rather roughly. According to the Shapiro-Wilk W tests, one cannot reject the null hypothesis that both the empirical and the simulated logarithmic phase durations for this stimulus condition are drawn from a normal distribution (W = 0.989, p = 0.540 and W = 0.992, p = 0.975, respectively). In the Chains model, short phases are rendered improbable by the self-excitation term, which is least adapted when an organisation first becomes dominant. The long tail of the distribution is caused by the noisy switching process that can sometimes result in the system getting stuck for rather long periods of time in one state.

### First phase choice

**Figure 8:**
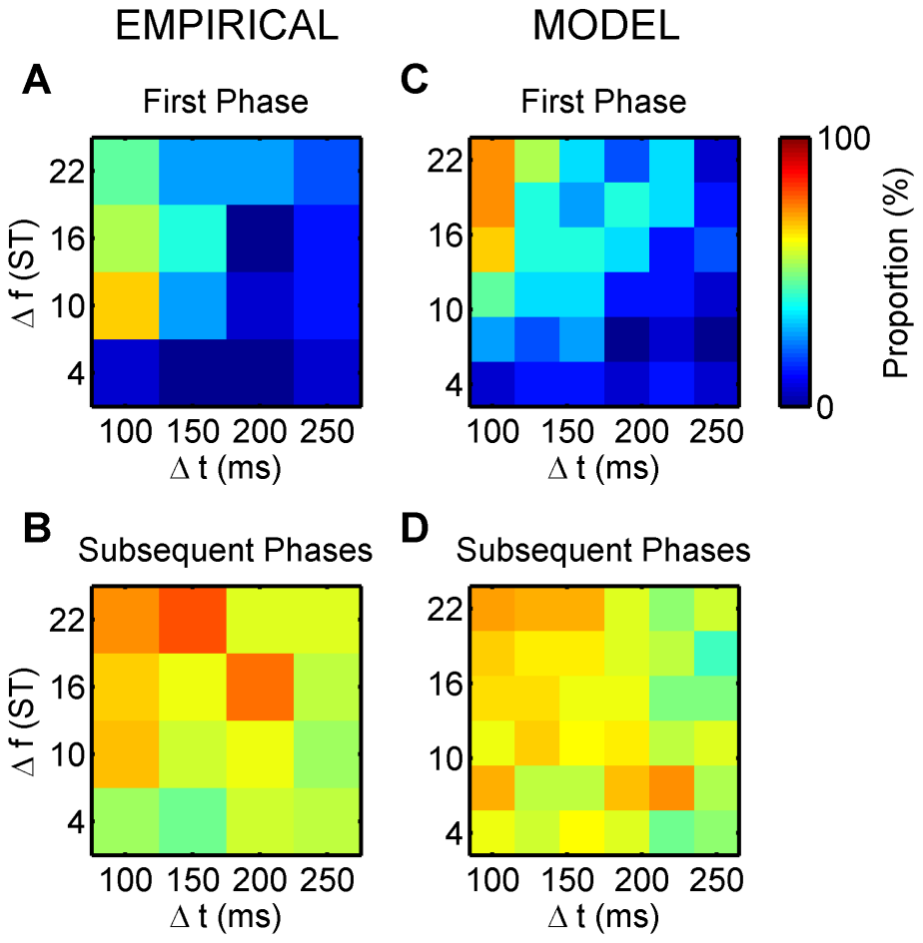
Proportion of time spent in the segregated organisation. A) An image displaying the proportion of experimental subjects (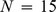) that reported hearing segregation first for the 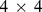 combinations of the stimulation parameters. B) An image displaying the proportion of time spent perceiving the segregated percept after the first perceptual phase has ended. C), D) The results from the Chains model (15 simulations) depicted in a 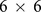 grid of the same parameter space corresponding to those presented in (A) and (B), respectively. Colour calibration of proportions (in %) is shown at the upper right corner.

The initial probability of linking events to form chains is determined by a combination of temporal proximity and similarity (rate of feature change from one event to the next), as shown by Cochran's Q tests on all conditions for both the empirical and the model data (Q = 56.669, df = 15, p<0.001, and Q = 102.608, df = 35, p<0.001), and by pair-wise Cochran's Q tests comparing the extreme conditions (see below). Temporal proximity favours integration, hence the first chain built is most often ABA−←. Both human listeners and the model have a bias towards first phase integration, as shown in Figure 8, top row. Figure 8A shows the proportion of instances in which experimental participants reported segregation as their first response as a function of 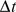 and 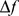. The corresponding results from the model are provided in Figure 8C. When 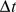 is short and 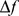 is large, listeners readily perceive a segregated sequence and take a long time to report integration. Conversely, when 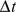 is long and 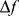 is small, they readily perceive an integrated sequence and take a long time to report segregation. Cochran's Q test of the perceptual reports showed that participants' first-phase choices significantly differ between these two parameter combinations (Q = 4.500, df = 1, p<0.034) as well as between small 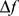 and large 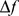 at short 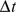 (Q = 4.500, df = 1, p<0.034). In the Chains model, this is determined by the probability of being able to form the links in the chains belonging to the integrated and segregated organisations, respectively. For example, when 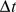 is short and 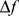 is large, the sequence contains abrupt changes in stimulus features, which make it less likely (though not impossible) that successive events will be incorporated into a single, integrated chain [75]. In this region of the feature space segregation is found first in a considerable fraction of the model runs (73.3%, significantly higher than in conditions with short 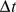 and small 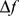 (bottom-left corner), with long 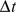 and small 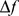 (bottom-right corner), and with long 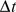 and large 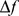 (top-right corner); Q = 10.000, df = 1, p<0.002, Q = 8.333, df = 1, p<0.004, and Q = 10.000, df = 1, p<0.002, respectively), similar to the reports of human listeners.

### First phase duration

**Figure 9:**
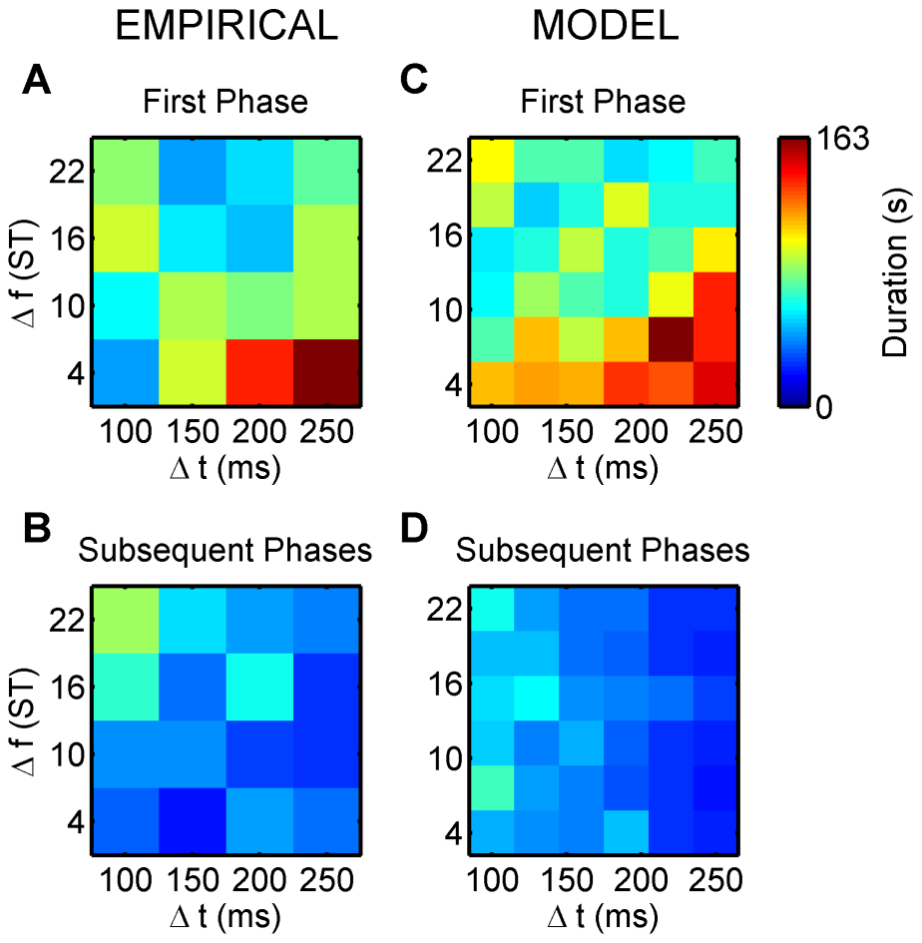
Durations of all perceptual phases. A) An image displaying the group-average (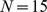) durations of the first perceptual phases, as reported by experimental subjects 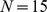 for the 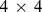 combinations of the stimulation parameters. “Integrated” and “segregated” phases were analysed together. B) An image displaying the mean durations of the perceptual phases subsequent to the first phase. C), D) The results from the Chains model (15 simulations) depicted in a 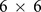 grid of the same parameter space corresponding to those presented in (A) and (B), respectively. Colour calibration of phase durations (in s) is shown at the upper right corner.

Figure 9 (top row) plots the mean durations of the first perceptual phases (“integrated” and “segregated” combined) for the experiment and the model. The ANOVAs showed significant interaction between 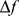 and 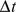 for the empirical data and a significant main effect of both 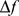 and 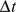 for the simulated data (F(9,126) = 3.733, p = 0.004, ε = 0.564, η^2^ = 0.210, F(5,70) = 8.480, p<0.001, ε = 0.613, η^2^ = 0.377, and F(5,70) = 2.023, p = 3.013, ε = 0.699, η^2^ = 0.177, respectively). The longest first phases coincide with the extreme conditions, small 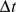, large 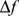 (top-left corner) and small 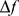, large 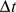 (bottom-right corner), respectively. The integrated first phases in this latter region of the feature space are particularly long (>120 s and significantly longer, according to Tukey's HSD tests, than with more balanced parameters in the centre of the grid; df = 126, p≤0.049 for the empirical results, df = 70, p≤0.011 for 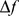 and df = 70, p≤0.048 for 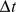 in the model simulations). This is largely due to the fact that under these conditions, the segregated chains are very difficult to discover since when 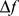 is small and 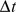 is large, the rate of stimulus feature change is very small, hence the probability that an event can be excluded from the building chains, which is necessary to discover A−← and −B−−←, is very low. In addition, when 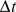 is large, the time between successive B sounds is especially long (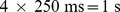), so the opportunity to form the B−← chain actually only occurs infrequently, thus it takes a long time to form the links necessary for building the −B−−← chain. Often, A−← *does* form in this corner, albeit after a long period. For example, at 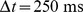 and 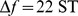, the A chain formed in all 15 model runs, after an average delay of 39 s. However, without the −B−−← chain, the A−← chain cannot overpower the integrated chain and dominate in the dynamical competition because its success rate is lower than that of the ABA−← chain (2 predicted events per cycle as opposed to 3). In the opposite (top-left) corner, the probability of discovering the ABA−← chain is low, but here temporal proximity helps somewhat in its discovery, whereas in the bottom-right corner, temporal proximity favours the integrated percept.

### Subsequent phases: Mean proportions of segregation

The strong tendency to report integration first for the majority of the parameter space results from the relative ease with which the integrated chain can be built in comparison to the segregated chains. However, once all three chains are built, they persist, and the competition becomes more balanced. Figure 8B shows the proportion of time that the segregated percept was experienced during the phases subsequent to (and excluding) the first phase. Figure 8D presents the results obtained from the model. They are very similar; the majority of the space reflects a more balanced competition (i.e., segregation reported roughly 50% of the time; light green shades in the colour map). ANOVA tests found no significant effect of the parameters on the overall proportions of the segregated percept (F(3,42) = 2.441, ε = 0.835, p = 0.90; F(3,42) = 0.950, ε = 0.713, p = 0.403; and F(9,126) = 1.729, ε = 0.589, p = 0.135 for the factors 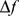 and 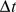 and their interaction, respectively). Similarly, in the model data, neither the main effect of 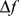, nor its interaction with 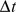 were significant (F(5,70) = 0.684, ε = 0.693, p = 0.586, and F(25,350) = 0.700, ε = 0.337, p = 0.698, respectively). Besides the overall balance in competition, a gentle diagonal gradient is apparent across both images (from which only the decrease in the proportion of segregated phases with increasing 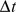 reached significance, F(5,70) = 2.905, ε = 0.650, p = 0.041, η^2^ = 0.172), such that segregation is more prevalent in the top-left, whereas integration is more prevalent in the bottom-right corner. In Chains, the reason for the continuing influence of stimulus features on perceptual dominance during subsequent phases stems from the rediscovery term; the likelihood of rediscovering a particular pattern enters into the dynamics of the model via the 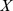 state variables.

### Subsequent phases: Mean phase durations

The mean proportion plots in Figures 8B and 8D show that a balanced competition is established once all three chains have been discovered. Balanced competition leads to more frequent switching [20], which means that the durations of phases subsequent to the first phase are considerably shorter on average. The mean durations for the subsequent phases are plotted in Figures 9B and 9D for the experiment and model, respectively. The experimental and model durations span a range of values with similar orders of magnitude (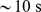). Phase durations generally increase as 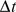 decreases (F(5,70) = 14.876, ε = 0.610, p<0.001, η^2^ = 0.515 for the model data, with a strong tendency in the experimental data, F(3,42) = 3.076, ε = 0.605, p = 0.068). No other effect or interaction was significant either for the empirical or the model data. The longer durations of the segregated phases in the region of the parameter space with small 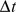 and large 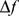 (top left corner) are the result of additional input via the 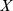 state variables to the segregated chains; i.e. a higher rediscovery rate for the segregated chains.

**Figure 10:**
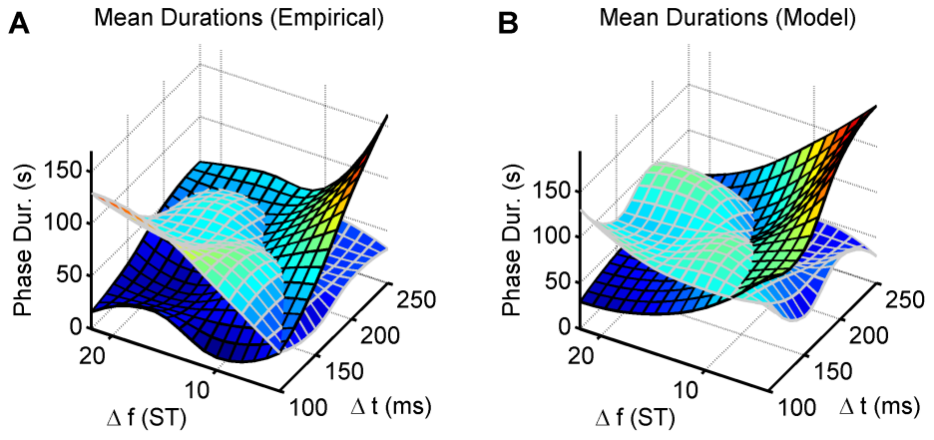
Durations of integrated and segregated phases. A) Surfaces showing the group-average (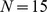) mean perceptual phase durations for integrated and segregated phases (cells outlined in black and white, respectively) as reported by listeners 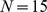. The first and subsequent phases were analysed together. B) The corresponding results obtained from the Chains model (15 simulations). Both surfaces were based on a 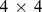 grid of the stimulation parameters (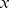 and 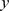 axes). Phase durations are calibrated in seconds on the 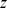 axis.

In Figure 10 the distribution of phase durations for integration and segregation is displayed separately as two intersecting surfaces for the experimental and model data. The first phase and subsequent phases are now combined. Qualitatively, the results for the model (A) and experiment (B) are very similar: the “integrated” and “segregated” duration surfaces show an exponential-like decay along the same diagonal gradient, but in opposite directions, such that they reach maxima in opposite corners and intersect in the middle. As expected from the discussion thus far, the longest integrated phases occur when 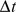 is maximal and 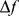 is minimal; the segregated durations are longest when the parameters fall at the opposite extreme. Viewing this figure we can also see that consistent with recent refinements to Levelt's second proposition [76], we find that *changes in stimulus parameters mainly affect the dominance durations of the dominant percept*.

### The time course of the probability of segregation

**Figure 11:**
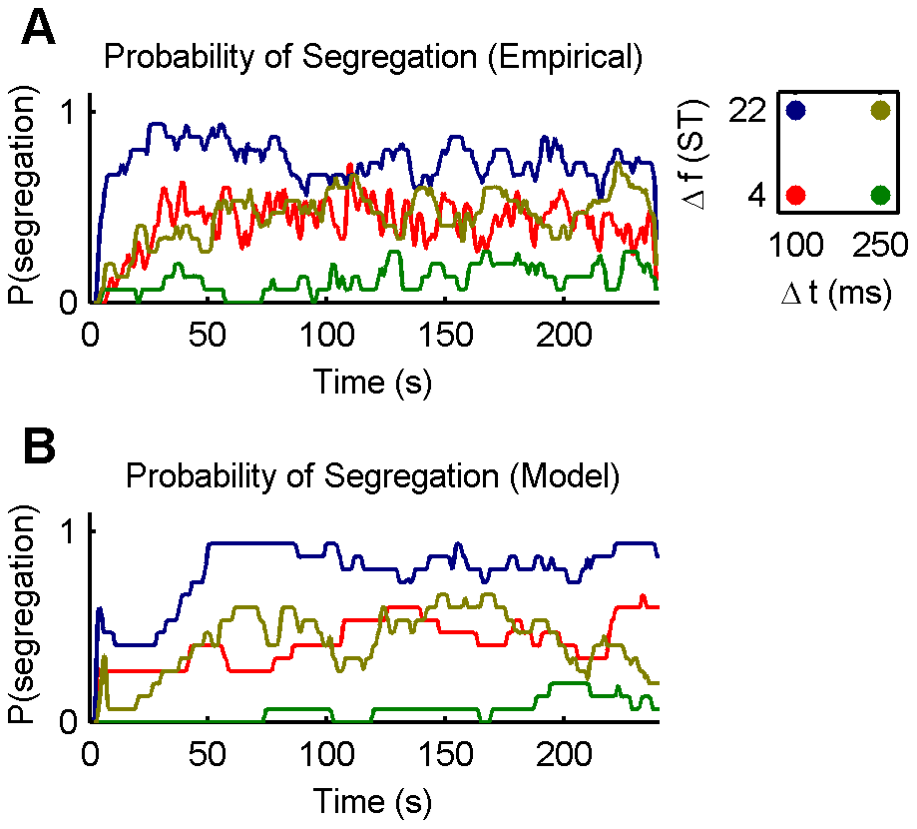
The time course of the probability of segregation. A) Four curves showing the group-average probability by which listeners (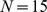) reported hearing the segregated percept at various times during a trial. The parameter combinations for each coloured curve are shown on the side map. B) The corresponding results from the Chains model (15 simulations). The probability of the streaming percept is always zero at the onset of the stimulus train as there is a delay to the first reported/modelled percept. This does not mean that listeners necessarily report (or that the model would find) the integrated percept before the segregated one (i.e., the probability of the integrated percept is also zero at the onset of the stimulus train).

In classical streaming experiments a great deal of attention has been paid to the “build-up” of streaming, with the notion that initially, a new stimulus sequence is perceived as integrated (the “default” organization) while segregating two or more streams requires gathering evidence from cues promoting separate grouping of subsets of sounds [3]. Results were typically presented by plotting the probability of segregation as a function of time for various combinations of 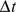 and 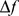 (see, e.g., [37], [77]). Figures 11A and 11B follow this procedure for the experimental and model data, and include a set of curves corresponding to the four extremes in the parameter space, i.e.,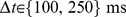, 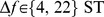. The trends are similar for the experimental and model data.

For 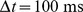 and 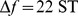 (blue curve; top-left corner of the parameter space), the probability of reporting segregation quickly rises to a large value after the onset of the sequence. In Chains, this reflects the fact that the chains that make up the segregated percept are easily built and, once active, suppress the building of the integrated chain. At the opposite corner of the parameter space, 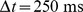 and 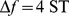 (green curve), both in the experimental and model data, the probability of reporting segregation rises slowly and reaches an asymptote at a rather low value. Note that this does not mean that some participants never perceive segregation, but that segregation is not experienced at the same time by all participants. In the model, the slow rise in the curve represents the fact that the integrated chain is discovered early and suppresses the building of the segregated chains. In the other two corners of parameters space (corresponding to the red and yellow curves), neither integrated nor segregated is favoured. As a result, the rate at which the probability of reporting segregation rises falls between the two extremes just mentioned, and the asymptotic values are close to one half, meaning that, on average, integration and segregation are roughly balanced.

**Figure 12:**
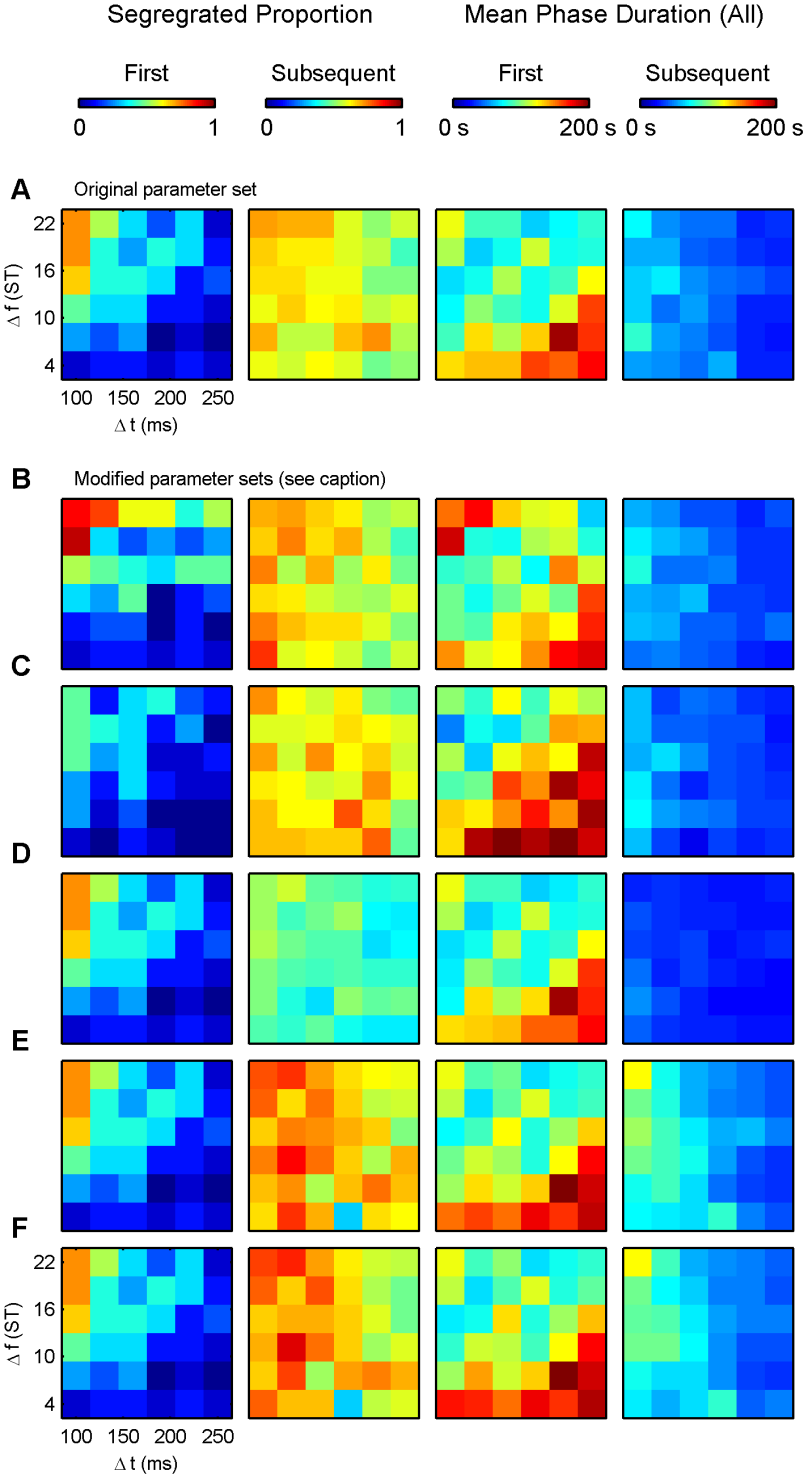
Illustration of the role of some model parameters. The left columns show the proportion of time spent in the segregated organisation separately for the first and subsequent phases, while the right columns display the durations of all perceptual phases (again, in separate columns for the first and subsequent phases). A) Results obtained with the original parameter set, specified in Tables 1 and 3. These charts are identical to panels C) and D) of Figure 8 and the same panels of Figure 9, respectively. B) Chain building parameter 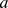 is changed from 0.00015 to 0.00075. Increasing the effect of rate-of-change on the inclusion probability renders it more difficult to form the ABA chain and thus the segregated percept is more prominent (especially with small 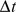 and large 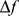 in the first phase and small 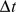 and 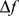 in subsequent phases). C) Chain building parameter 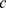 is changed from 0.0055 to 0.0035. Decreasing the probability of skipping over auditory events promotes the chains of the integrated organization, especially when rate of change is small. D) The weighting coefficient of success rate 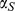 is changed from 3.8 to 3.9. As the number of successful predictions a chain makes in unit time have a larger effect on its excitation, the integrated percept (with the highest success rate) is more dominant in subsequent phases than with the original parameter value. E) The weighting coefficient of the inhibitory signals towards the excitatory populations 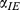 is changed from 8.1 to 8.2. The resulting increase in the effectiveness of collisions in lowering chain excitation is manifested by a small bias towards the segregated percept (whose corresponding chains incur fewer collisions) in subsequent phases. Further, switches are less probable, i.e. phase durations are higher, when inhibition is more efficient in suppressing momentarily non-dominant chains. F) The weighting coefficient of noise 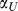 is changed from 3.4 to 3.0. As noise is responsible for the perceptual switches, decreasing its contribution to the excitation of the chains lengthens subsequent phases (especially when 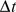 is small). Note that adjusting the weighting coefficients of the dynamical state variables in panels D), E), and F) has no influence on the first phases (that is governed exclusively by chain discovery). Colour calibration is shown on top.

The effect of the model parameters on the results obtained are illustrated on Figure 12. By changing the chain building parameters, it is possible to influence the balance between the two organizations. The integrated chain needs to connect different events, while the chains corresponding to segregation must omit events. The cost of these operations are set by parameters 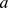 and 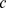, respectively. The weights of success rate, inhibition, and noise in determining the change in the excitations influence both the balance of the two percepts in subsequent phases and the stability of the competition.

## Discussion

The data show that the Chains model simulates the perceptual behaviour of human listeners in streaming experiments very well, accounting for both the contents and the dynamics of perceptual awareness. To our knowledge this is the first computational model of auditory streaming that is able to do so. Influenced by the notion of a perceptual decision, argued very persuasively by Bregman [3], previous computational models (e.g. [78]–[82]) all focussed essentially on the first perceptual phase, and the time course and feature dependence of the probability of segregation. None of these models is able to account for perceptual switching between integration and segregation as they all have one stable, fixed, feature-dependent attractor. Moreover, none of these models addressed the fundamental question we have identified here and in previous theoretical work [1], [42] regarding the nature of the attractors and how they are discovered. Instead all previous models of auditory streaming have assumed two possible decisions; i,e., integration or segregation. Thus, in general, they do not really touch upon the notion of auditory objects as temporally persistent representations built up from regularities detected in the sensory input, as defined by Winkler et al [1], and they do not have much to say about the principles underlying perceptual organisation. The exception is the Kalman-filter model of Elhilali & Shamma [79], which captures the notion of temporally persistent representations of regularities in the filters that are derived from the input sound sequence, and also uses the predictions made by the filters to refine these representations. Thus far this model has not been extended to simulate perceptual switching; nevertheless there are some important commonalities between their approach and the model we propose here.

Although similarities between the perceptual switching behaviour in visual and auditory bistability experiments has previously been reported [26], models of visual bistability cannot be trivially applied to auditory streaming. The most obvious reason is that in audition the stimulus is experienced over time, and all parts of the object are not simultaneously present, whilst in binocular rivalry (the most frequently simulated example), there is an assumption that the stimulus is continuously present in its entirety. Therefore, while being inspired by the attractor dynamics approach employed by a number of models of visual multistability (e.g. [20], [22]), the Chains model, by necessity, extends those models in two important ways. Firstly, Chains shows how attractors can be discovered, and secondly, how the nature of the competition between them ensures the simultaneous emergence of compatible proto-objects into dominance (perceptual awareness). Chains thus accounts for the emergence of perceptual organisations, each of which can contain an arbitrary number of perceptual objects. It should be noted that although the model is currently expressed in a rather abstract way, it has been formulated with a view towards understanding perceptual processes as they occur in the brain.

We shall now review Chains in the light of previous theoretical and experimental work, noting points where the model should be extended to cover a wider set of perceptual phenomena and more ecologically valid stimuli. Proceeding in a “bottom-up” fashion, we consider: what might constitute an elementary “event” in physiological terms; how the events are grouped into chains; how the build-up and failure of chains relate to the time course of streaming; and the nature of the dynamics that ensues between concurrently active (closed and predicting) chains. Finally we conclude with a summary of the theoretical contributions of the model.

### Events

The basic elements handled by the Chains model are discrete sound *events*. We assume that event decomposition is largely based on instantaneous cues of sound segregation [3], [83] (such as common onset and harmonicity) which are processed before the sequential regularities modelled in Chains are detected. The series of auditory ERP responses reflecting the detection of event onsets as they are triggered by abrupt acoustic changes [51], [84] precede and overlap the ERP marker of sound segregation by instantaneous cues (the object-related negativity; ORN [83], [85]), both of which precede prediction-based detection of sequential deviance (MMN [84]). There is evidence showing that deviations occurring 300 ms or farther from the last N1 are not flagged as prediction errors by MMN [86]–[88]. This suggests, as assumed in our model, that event decomposition is tied to event onsets and it is a prerequisite for extracting sequential regularities from a sequence. The way in which continuous sounds can also be included in this time-localised framework requires further consideration.

### Event similarity

We posit that it must be possible to measure similarity relationships (featural distance) between any two events. The feature we have made use of in this modelling study is frequency, for which a natural perceptual measure of distance is the frequency ratio, measured as an absolute frequency difference on an octave scale. Although we have not included other acoustic features in the model presented here, we assume that all features (e.g., loudness, location, pitch, etc) are bound together into a single event representation even outside the focus of attention [89]–[92]. It is therefore necessary for simulating the perceptual organisation of complex sounds to formulate distance measures that encompass all the features of relevance. This is outside the scope of the current model, but depends upon the reasonable assumption that relationships rather than absolute features form the basis for the perception of similarity [43]. Therefore, a full model of auditory scene analysis will need to include two-way links between the sequential grouping processes (modelled by Chains) and the assumed pre-processing steps that establish the event components (instantaneous grouping) and inter-event relationships (rate of change).

### Chains

Once events are extracted, there remains the question of how they come to be linked. There is compelling evidence that simple, chain-like rules are encoded within the brain [84], [93] and they can be studied using the MMN ERP component elicited in response to rule violations. Evidence from MMN studies has demonstrated that several rules can be maintained concurrently even outside the focus of attention [53], [94], [95], and, in keeping with our model, that the length of a stored pattern is restricted to a few items, or in total duration [4], [96]–[99].

A legitimate objection at this point is that Chains is overly restrictive, in that it can only represent rules in the form of deterministic periodic patterns, whereas, in fact, MMN can be elicited by a host of non-periodic stimuli [93]. Furthermore, auditory bistability can be elicited by non-periodic patterns; e.g. with randomly jittered tones having a predictable distribution [71]. In response, we note that the looping chain representation was chosen initially, because it most naturally accommodates the format of the repeating tone stimuli used in auditory streaming experiments. However, periodic sequences are not the essence of the model, which consists of the following: (i) the parallel encoding of regularities; (ii) predictions based upon those regularities; and (iii) competition amongst the predictors at the level of individual (local) events, resulting in the spontaneous (global) emergence of stable organisations. A more comprehensive version of the model would employ a wider repertoire of predictors, which encode non-periodic sequential predictions (e.g., whenever A occurs, B follows 100 ms later), relative and second-order changes (e.g., frequency changes that alternate in sign [94], or ‘the higher the pitch the lower the intensity’ [100]), statistical distributions, and so forth. The model we present here provides a flexible framework for these extensions.

A notable aspect of the chain building process is the probabilistic exclusion term. This is important, as without being able to skip over events, the model would not be able to discover patterns consisting of non-adjacent events embedded within sequences [101]. Detecting embedded regularities is clearly ecologically relevant; for example, in conversations the utterances of different speakers typically interweave in the ongoing interaction. It is also possible that the exclusion function is actually time varying. This is suggested by neurophysiological experiments showing the development of differential suppression, i.e. the gradual reduction in responses to one or other of the tones in a streaming sequence [37], [102], [103]. Clearly the formation of links between non-adjacent events would become more likely as the activity associated with intervening events becomes weaker; and this could be modelled by a time-varying exclusion function.

The exclusion function as presented here introduces a simplification. The first condition that prevents the exclusion of an event that matches any of the events already included in the building chain is overly restrictive. If it is removed then the model discovers other repeating patterns, and other organisations, e.g. {AB−−← and −−A−←}, {−BA−← and A−−−←}. These organisations are not well described by the classic integration/segregation distinction employed in most streaming experiments to date. In our experiments [12], [14], [41], [71], [72] we found that if participants are not instructed with an implied forced choice and are told they may sometimes hear other organisations that include both A and B tones in one stream as well as a separate stream containing only A's, then they report hearing these other organisations too, and do so with a probability which can be rather high (up to ca. 30% with some stimulus configurations). Similar results have also been reported in visual bistability [76]. We are currently conducting an experiment to tease out the full range of patterns that participants perceive. Therefore, and also in order to facilitate comparison with the classical auditory streaming literature, we did not explore this issue here: the experimental data reported in this paper was acquired using the traditional ‘exclusive choice’ instruction set.

The other simplification of the model presented here is the deletion of chains whose predictions fail. As noted previously this is overly restrictive, and there is evidence from MMN studies for the persistence of regularity representations through multiple consecutive deviant events (e.g., [53]) and even for a dormant (currently inapplicable) state of the representations, which can, however be reactivated (brought back to an active state) by a single “reminder” event [54]. Thus we suggest that in the future, chains should be penalised by weakening rather than removing them. Such soft penalties will require the introduction of further parameters, for which we have insufficient experimental data at this stage. Nevertheless, relaxing the current ‘death penalty’ is necessary for enabling the model to form representations of distributions and to track perceptually acceptable changes and variations in the features of auditory objects. Within the larger picture, this extension of the model can be regarded as a step towards accounting for the observed stability of perception.

One further simplification of the model, when compared to the auditory streaming experiment, is that whereas each simulation is started afresh, human listeners may retain some information from preceding stimulus blocks. Specifically, if a proto-object has been formed in a stimulus block, it may still be available at the beginning of the next one following the typical short breaks used in psychophysics experiments, including our own. If at least some of the sounds are identical between the stimulus blocks, as was the case in our experiment, then these act as reminders, activating the proto-objects including the common stimuli. As it has been mentioned above, reactivation of auditory regularity representations (proto-objects) was demonstrated in both behavioural and ERP studies, even after a silent interval of 30 s (for a review, see [54]). The carry-over of proto-objects from one 4-minutes long stimulus sequence to the next benefits the perceptual alternative that would have otherwise been discovered only later during the new stimulus sequence: in most cases, the segregated percept. Indeed, Snyder and colleagues found a significant carry-over effect from one trial to the next in a similar study [104]. In our context, this claim can be tested both experimentally, by changing the parameters of the tones between the sequences, and in the model, by retaining the chains from the previous condition when simulating the next one. However, this raises questions about chain generalisation that are beyond the scope of the current paper.

### Dynamics and multistability

Tone sequences of even a modest complexity, such as the ABA−ABA−… tone pattern employed in this work, contain within them many different embedded patterns. In the Chains model, each pattern discovered is encoded as a chain. It is important to recall that “conflict” between two chains does not mean that they predict different tones at the same moment; on the contrary, they each predict the *same* tone, but assign it to a different causal pattern. Chains are mutually inhibitory to the extent that their predictions collide, and this in turn leads to the formation of stable subsets of chains (organisations) whose predictions are complementary. The presence of noise in the dynamics means that an organisation cannot dominate perception indefinitely; instead, it will eventually collapse, and during the momentary instability that ensues, a different set of chains will emerge as the dominant perceptual organisation.

Once a chain forms, its prominence is governed by a set of dynamics which interacts with the dynamics of the other chains. The variables that steer the dynamics (listed in Table 2) correspond to running averages of event-related incidents, such as successes and collisions. The proposal that these variables could in some way be represented in the brain gains credibility when one considers that they require only local computations [42], both in terms of time (computation is memoryless, except for a leaky integration), and in terms of representation (computation is local to a chain; there are no global interactions or parameters). It could be argued that the chain matching operation required for computing the rediscovery term (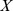) is not a local computation, and, in the rather abstract algorithmic version of the model presented here, this is true. However, we would argue that the instantiation in the brain of such a process is likely to be more straightforward, and involve only local computations that simply equate to short term learning which (temporally) strengthens the links on the neural pathways that represent the chain in ‘synfire chain’-like structures [105].

Competition amongst chains is implemented along the same lines as a number of models of visual bistability [20]–[23]: cross-inhibition, self-adaptation and noise. The relative importance of these factors, and the precise way they interact, has been the subject of much discussion [20], [22], although there is a general consensus that all three must be present. Cross-inhibition and adaptation without noise leads to periodic deterministic dynamics, and although cross-inhibition and noise without adaptation (i.e., a noise-driven attractor model [20]) is sufficient for random phase alternations to occur, detailed analysis of the phase statistics provides evidence for an adaptation process in addition to the noise [106], or a noisy adaptation process [22]. Specifically, it has been argued that a relatively slow adaptation process accounts for weak correlations in consecutive perceptual phases of the same type [19] and the gamma-like shape of the phase distribution [20]. The Chains model includes both noise and adaptation (see the “Chain Dynamics” section in Models). Disabling noise abolishes switching altogether, whereas disabling adaptation does not, although phase durations are substantially lengthened as a consequence, and their distribution reverts to an exponential shape (results not shown).

The major difference between the dynamics in Chains and other models lies in the nature of the cross-inhibition. Because models of visual bistability generally use predefined attractors, either cross-inhibition between the attractors or global inhibition is hardwired, and inhibition acts continuously. In Chains, we discover the attractors and their points of conflict (i.e. attributing events to different causes), and inhibition only occurs between conflicting attractors. Inhibition is thus dynamic, and its time course is determined by the occurrence of conflicting events.

The most important factor in ensuring the emergence of perceptual organisations and perceptual switching is the balance of the success and collision variables, determined by 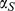 and 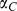, respectively. Specifically, we found that increasing 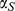 promotes integration, whereas increasing 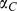 (or 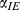) promotes segregation. This is reasonable in light of the fact that: (i) the ABA−← chain receives 3 successes per cycle (compared to the 2 and 1 successes per cycle of the A−← and −B−−← chains) so increasing 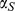 favours ABA−← (integration), and (ii) the A−← and −B−−← chains together exert a large degree of inhibitory influence on ABA−←, so increasing the inhibitory weights favours A−← and −B−−← (segregation). (For an illustration, see Figure 5.) In summary, a specific balance of excitation, inhibition and adaptation is required to yield bistable switching, and a distribution of perceptual dominance that matches human experience.

### “Build-up” of streaming

The model was not explicitly tuned to simulate the assumed build-up of streaming. However, by correctly simulating the characteristics of the first phase, including the initial perceptual decision (first phase choice) and first phase duration, the model also simulates the typical build-up patterns reported in the literature (for example, see [37], [41], [77], [107]).

The first phase is dominated by integration for most sets of parameters. This occurs because temporal proximity favours the discovery of the ABA−← chain, while discovery of the A−← and −−B−−← chains requires skipping over intervening events. On the other hand, similarity favours the A−← and −B−−← chains and thus when the A and B tone features are very dissimilar, there is a chance that the influence of similarity will override temporal proximity, and the A−← chain will be discovered first (followed closely by the −B−−← chain, thanks to the 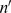 term in the exclusion function). However, because of the way the data is plotted (i.e. as a probability of reporting segregation), and because the first point is always zero (i.e. with no sounds the probability of segregation is zero), plots that display the probability of segregation averaged across participants as a function of time can give the misleading impression of a gradual ‘build-up’ rather than an initial perceptual decision in favour of segregation or integration.

Behind the morphological similarity between the simulation data and the well-known results supporting the classical notion of the build-up of streaming [3], [77], there is a fundamental theoretical difference between our model and previous work. Chains does not make any assumption about a default sound organization, nor does it “gather evidence” only for segregation. “All chains are equal” in how they are discovered. The time necessary for their discovery depends only on the parameters of the model and the actual sound sequence. Thus, properly speaking, our model suggests that there is no “build-up” of streaming; there is a build-up of chains, whether they represent the integrated or the segregated percept.

In auditory streaming experiments, the special character of the first perceptual phase has been previously noted [41], [108], with the influence of stimulus features being far stronger during the first phase than subsequent phases. In their experiments, Hupé and Pressnitzer [108] showed that first percept choice and the duration of the first phase (inertia) were independent. This is consistent with our model in which the first percept is determined by which pattern is easiest to discover, while first phase duration is largely determined by how long it takes the system to discover other patterns and pattern combinations.

### Conclusion

The principal contribution of the Chains model is to show that a process with two parallel stages, pattern discovery, and competition between incompatible patterns, can account for both the contents (perceptual organisations) and the dynamics of human perception in auditory streaming experiments. In this, our model is compatible with Bregman's theoretical framework [3]. However, we suggest an alternative to Bregman's specific proposal that auditory perception works by ‘accumulating evidence’ in favour of some perceptual decision. Instead we suggest that perception emerges from a process that creates, possibly many, alternative interpretations of the sensory scene in parallel, and samples these interpretations with a probability that is related to their likelihood (ease of discovery). The proto-objects (chains) that form and compete with each other do not necessarily all enter conscious awareness, and those that are incompatible cannot do so simultaneously. Proto-objects that win the competition become the auditory objects of perception. Thus if the stimulus is rather short or if it changes, then there may be time for only the most likely proto-objects to be perceived. This may explain our everyday experience that, in general, perception tends to provide an unambiguous and stable interpretation of the world.

Although the dynamics in the model are governed by similar factors employed in a number of visual models of bistability, the model we present here goes beyond previous work in proposing mechanisms by means of which competing representations can emerge, rather than being predetermined. This allows us to also account for the qualitative differences between first phase and subsequent phase behaviour [41]. Perhaps it is this ability to discover and simultaneously represent a number of different interpretations of the world and to flexibly switch between them that underlies the robustness of natural perception.

## Supporting Information

Audio S1
**The stimulus sequences used in the auditory streaming experiment.** Each sequence is a 4-minute long cyclic repetition of an isochronous ABA– pattern, where A and B are pure tones of different frequencies and – denotes a silent gap. The names of the sound files specify the combinations of 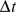 and 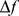 stimulus parameter values for each tone sequence, with the former one referring to the frequency difference between the tones (A tones were kept constant at 400 Hz and the frequency of B tones was varied according to 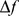) and the latter denoting the onset-to-onset time interval.(ZIP)

Protocol S1 **The source code of the Chains model.** The model is implemented in C, with a Matlab interface. After compilation, it can be used to run model simulations with arbitrary repetitive pure tone sequences. A brief tutorial and example scripts are included. The source code is also available for download at http://sites.google.com/site/chainsmodel/.
(ZIP)
