# Supplementary material for: Modelling the Emergence and Dynamics of Perceptual Organisation in Auditory Streaming
Source: PLoS Comput Biol. 2013 Mar 14;9(3):e1002925. doi: 10.1371/journal.pcbi.1002925 (PMC3597549; doi:10.1371/journal.pcbi.1002925)
Supplement: Protocol S1 — The source code of the Chains model. The model is implemented in C, with a Matlab interface. After compilation, it can be used to run model simulations with arbitrary repetitive pure tone sequences. A brief tutorial and example scripts are included. The source code is also available for download at http://sites.google.com/site/chainsmodel/. (ZIP) [file pcbi.1002925.s002.zip › CHAINS v2.0 User Guide.pdf]

CHAINS:
User Guide

version 2.0

sites.google.com/site/chainsmodel
rob.mill.uk@gmail.com

1. Quick Start 2
2. Nodes and Chains 5
3. Running the CHAINS Algorithm 10
4. Chain Dynamics 13
5. Advanced Data Types 22

1. Quick Start

Initial Setup

Adding paths

First, unzip the CHAINS-v2.0.zip file into a working directory. Then add this directory and all its subdirectories to the MATLAB path. This can be done via the main menu by following File → Set Path → Add with Subfolders.... Alternatively, it can be done by typing the following at the MATLAB console whilst in the root CHAINS directory:

```
addpath(genpath(pwd));
```

Compiling C/Mex source

The CHAINS algorithm itself is implemented in C. In order to use it from MATLAB, it must first be compiled[1]. This procedure need only be performed one time. To compile the C code, type the following at the MATLAB console:

```
compile_C
compile_Mex
```

If these routines complete without error, then CHAINS is ready to use.

Sound Sequences

ABA Sequence

ABA sequences consist of a repeating isochronous sound pattern A, B, A, –, where A and B are pure tones with different frequencies and the "–" is a silent gap equal in duration to one tone. CHAINS forms three chains in response to an ABA sequence. The first predicts all three tones (ABA−); the second predicts just the As (A−); the third predicts just the Bs (B−−−).

The two key parameters of an ABA sequence are the frequency difference between the A and B tones (Δf), and the interval between the onset of the A and B tones (Δt). These are given as the first two parameters to a function called eg_aba:

```
eg_aba(8, 150, 0);
```

This supplies an ABA sequence as input to CHAINS, where the frequency difference is 8 semitones (ST) and the onset-to-onset interval is 150 milliseconds. The third parameter, `0`, fixes the random seed at zero at the start of the run, to ensure that results can be replicated. Omitting the third parameter runs CHAINS with a randomly-chosen seed. Type `help eg_aba` for more detail. The routine generates a figure plotting the excitation of the three chains.

[1] MATLAB does not currently support compilation of Mex files on 64-bit platforms.

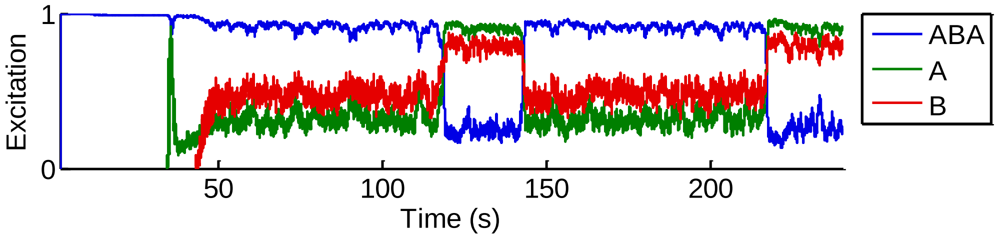

To return the excitations of the three chains, request an output argument. For example:

```
E = eg_aba(8, 150, 0);
```

This routine returns a 3 x *n* matrix, E, where *n* is the number of time steps the routine runs for. Each column corresponds to a time step of one millisecond. The first, second and third rows of the matrix correspond to the ABA, A and B chains, respectively. Where there is no chain—either because it has not yet formed or because it never forms—the matrix is padded with `NaN`s.

Arbitrary Frequency Sequence

The second piece of example code generalises the `eg_aba` function above, allowing the user to input an arbitrary sequence of tones into CHAINS and plot the results. The function is called `eg_freqs`. To input an alternating tone with a rate of 10 Hz and Δf equal to 8 ST, for example, type the following in the MATLAB console:

```
eg_freqs([0 8], [100 100], 0);
```

The first and second parameters specify the tone frequencies in semitones[2] and the onset-to-onset time in milliseconds, respectively. The third parameter is the random seed. Type `help eg_freqs` for more detail. The output figure is shown below.

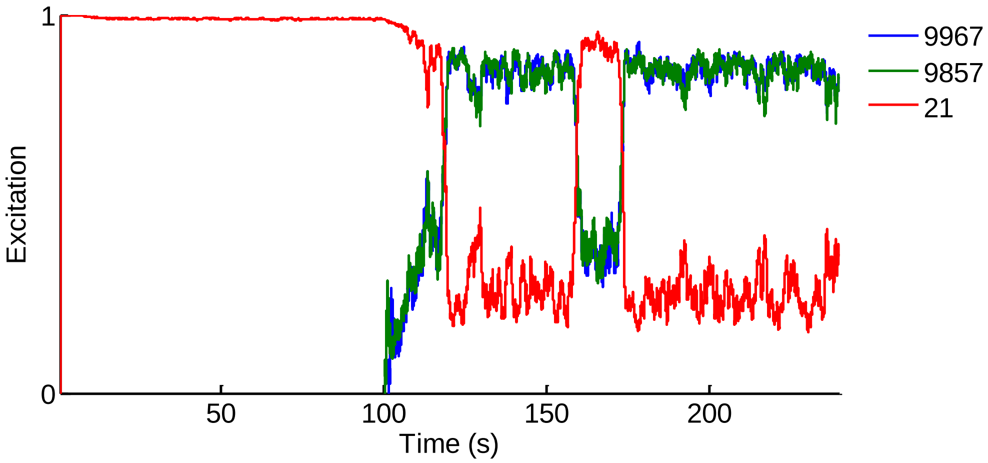

[2] Note that, by default, CHAINS uses only the frequency difference between the tones (in semitones).

Because the number of chains that forms in response to an arbitrary sequence is not known in advance, the chains are labeled with a unique numeric identifier[3] (ID). These IDs are plotted in the figure legend, and a description of the chain is printed in the console for each ID. The output to the console in this case is:

```
ID        |  Flags     |  Phase     |  Cycle  |  Nodes
    9967  |  ---LPC-   |         0  |    694  |  [0]--(200)-->
    9857  |  ---LPC-   |       100  |    699  |  [8]--(200)-->
      21  |  ---LPC-   |         0  |   1199  |  [0]--(100)-->[8]--(100)-->
```

There are three chains. The first is tone "A" repeating every 200 ms; the second is tone "B" repeating every 200 ms; the third is an integrated chain "ABABAB...". CHAINS is able to find an integrated organisation consisting of AB alternating on its own, or an organisation consisting of two segregated chains, one containing As and the other containing Bs.

The excitation values for these chains can also be recovered by requesting an output argument:

```
E = eg_freqs([0 8], [100 100], 0);
```

[3] Unique IDs are generated by incrementing an internal counter. This counter is reset when `clear all` is typed at the MATLAB prompt. If the IDs in the example output shown in this document differ to those obtained when running the examples, it is likely that the counter started at a different value.

2. Nodes and Chains

Nodes

A **node** encodes a discrete sound event (**data**) followed by a pause. Nodes are created using node_new and can be printed to the screen using node_print. The example below creates a node called node1, which encodes the data 1000 and then pauses for 250 ms. The node details are then printed to the console.

```
% Create the node
node1      = node_new;
node1.data = 1000;
node1.wait = int32(250);

% Print the node
node_print(node1);
```

Nodes are copied using node_copy. For more details about the fields of the node structure, type help node_new. For help with the functions that manipulate nodes, type help node.

Chains

A **chain** essentially encodes a list of nodes (i.e. a temporal pattern of discrete sounds) with some additional details. Chains are created using chn_new and can be printed to the screen using chn_print. The nodes in the chain are stored as an array of nodes (see above) in the .nodes field. Modifying a chain by changing its fields directly is not recommended. Rather, the suite of functions designed to modify chains should be used. The important ones are now described below.

Creating and Printing an Empty Chain

The example below creates an "empty chain" called chn1, which contains no nodes. The empty chain is then printed.

```
% Create and print an empty chain
chn1 = chn_new;
chn_print(chn1);
```

Flags

Chains are interpreted differently depending on the how the **flags** (binary options) are set. All flags are set to zero (cleared) when a chain is first created. A list of flags is given in the table below, along with a description and the initial abbreviation used by `chn_print`.

Flag	Description	
input	chain encodes input sounds	`I`
open	chain grows with input	`O`
closed	chain no longer accepts input but must reform links	`X`
loop	chain is a loop (linear if flag is cleared)	`L`
predict	chain makes predictions	`P`
compete	chain competes with other chains	`C`
window	chain is windowed[4]	`W`

Flags are set or cleared using `chn_setflag` or `chn_clearflag`, respectively. To query which flags in a chain are set, using `chn_flags`. Examples of how these are used are given in the next section.

Adding a Node to a Chain

Nodes are added to a chain using `chn_addnode`. Before a node can be added to a chain, the "open" flag must be set. The example below creates chain, sets its open flag, and adds a single node "A".

```
% Create an empty chain and set it open to add nodes
chn1 = chn_new;
chn1 = chn_setflags(chn1, 'open');

% Add a node containing data 'A'
chn1 = chn_addnode(chn1, 'A');

% Print the chain
chn_print(chn1);
```

The output of this routine is

```
ID        |  Flags     |  Phase     |  Cycle  |  Nodes
       1  |  -O-----   |        0   |      0  |  [A]
```

The output shows that the chain consists of a single node "A", has a unique identifier "1", and has its open flag set "O".

[4] Linear input chains may contain a large number of nodes, in which case node-to-node comparisons between chains becomes computationally demanding. Enabling *windowing* disables comparisons between events that are more than two seconds in the past or the future.

Advancing a Chain with the Passage of Time

Chains are advanced using chn_advance. If the "open" flag of a chain is set, then the chain *grows longer* as time advances. In this way, alternating invocations of chn_addnode and chn_advance allow chains to be built up with an arbitrary pattern. The example below creates an empty chain, sets its "open" flag, adds the node "A", advances it 100 ms, adds the node "B", and then advances it another 50 ms. The chain is then printed.

```
% Create an empty chain and make it open to add nodes
chn1 = chn_new;
chn1 = chn_setflags(chn1, 'open');

% Add a node containing data 'A' and advance 100 ms
chn1 = chn_addnode(chn1, 'A');
chn1 = chn_advance(chn1, 100);

% Add a node containing data 'B' and advance 50 ms
chn1 = chn_addnode(chn1, 'B');
chn1 = chn_advance(chn1, 50);

% Print the chain
chn_print(chn1);
```

Printing this chain now gives

```
ID         |  Flags     |  Phase     |  Cycle  |  Nodes
       1   |  -O-----   |      150   |      0  |  [A]--(100)-->[B]--(50)-->
```

This chain will continue to grow in response to chn_addnode or chn_advance. To make the chain a "looping input" chain, we can use chn_setflags, chn_clearflags and chn_setphase as follows (after running the code above).

```
% Make chain looping input
chn1 = chn_clearflags(chn1, 'open');
chn1 = chn_setflags(chn1, {'loop', 'input'});

% Reset chain phase to zero
chn1 = chn_setphase(chn1, 0);
```

Printing this chain (using chn_print) now gives the following output, indicating the chain is at the start (phase = 0), is a loop (flag "L" set), and describes sound input (flag "I" set).

```
ID         |  Flags     |  Phase     |  Cycle  |  Nodes
       1   |  I--L---   |        0   |      0  |  [A]--(100)-->[B]--(50)-->
```

Chains are copied using chn_copy. For more details about the fields of the chain structure, type help chn_new. For help with the functions that manipulate nodes, type help chn. Note that many of the functions accept and return *lists* of chains (e.g., one can advance many chains at the same time).

Building the ABA Chain

The introduction to the basic chain functions given above is sufficient to explain how to build an “ABA–” input chain (or any other input chain). This code forms the core of the eg_ABA function described earlier. The example below builds a looping ABA chain with frequencies A = 0 semitones and B = 4 semitones, and an SOA of 100 ms.

```
% Create an empty chain and make it open to add nodes
ABAchn = chn_new;
ABAchn = chn_setflags(ABAchn, 'open');

% Add a node containing data 0 semitones and advance 100 ms
ABAchn = chn_addnode(ABAchn, 0);
ABAchn = chn_advance(ABAchn, 100);

% Add a node containing data 4 semitones and advance 100 ms
ABAchn = chn_addnode(ABAchn, 4);
ABAchn = chn_advance(ABAchn, 100);

% Add a node containing data 0 semitones and advance 200 ms
ABAchn = chn_addnode(ABAchn, 0);
ABAchn = chn_advance(ABAchn, 200);

% Make chain looping/input
ABAchn = chn_clearflags(ABAchn, 'open');
ABAchn = chn_setflags(ABAchn, {'loop', 'input'});

% Reset chain phase to zero
ABAchn = chn_setphase(ABAchn, 0);

% Print the ABA chain
chn_print(ABAchn);
```

Note that calling chn_advance on ABAchn advances its phase, but does not cause the chain to grow (because the “open” flag is cleared). For example, printing a version of the chain advanced by 200 ms using

```
% Print chain advanced 200 ms (phase at 200 ms)
chn_print(chn_advance(ABAchn, 200));
```

produces the output:

```
ID        |  Flags     |  Phase     |  Cycle  |  Nodes
       1  |  I--L---   |       200  |      0  |  [0]--(100)-->[4]--(100)-->[0]--(200)-->
```

And printing a version of the chain advanced by 450 ms using

```
% Print chain advanced 450 ms (one complete cycle; phase wrapped at 50 ms)
chn_print(chn_advance(ABAchn, 450));
```

produces the output:

```
ID        |  Flags    |  Phase    |  Cycle  |  Nodes
       1  |  I--L---  |       50  |      1  |  [0]--(100)-->[4]--(100)-->[0]--(200)-->
```

Notice the values of the “cycle” and “phase” fields, as reported by `chn_print`.

3. Running the CHAINS Algorithm

The previous section gave instructions on how to build an input chain up from scratch. The CHAINS algorithm is invoked using `chn_run`. The input to `chn_run` is the list of chains which are present at the *start* of a fixed time period. The output of `chn_run` is the list of chains which is present at the *end* of a fixed time period.

Running CHAINS

Running the CHAINS algorithm simply adds another step beyond what we have already seen in the previous section. The code example below creates an input chain consisting of 0 repeating every 150 ms. The CHAINS algorithm then runs for 240 seconds[5], starting with this one input chain. Note that `runopt` contains the options that CHAINS should use when running, which we set to default values in this example.

```
% Create an empty chain and set make it open to add nodes
inchns = chn_new;
inchns = chn_setflags(inchns, 'open');

% Add a node containing data 0 semitones and advance 100 ms
inchns = chn_addnode(inchns, 0);
inchns = chn_advance(inchns, 100);

% Make chain looping/input
inchns = chn_clearflags(inchns, 'open');
inchns = chn_setflags(inchns, {'loop', 'input'});

% Reset chain phase to zero
inchns = chn_setphase(inchns, 0);

% Run the CHAINS algorithm
runopt  = def_runopt;
outchns = chn_run(inchns, runopt);
```

In this example, the chains which are present at the end of the 240-second period are returned in `outchns`. (The output chains include the original input chain.) The output chains are also automatically printed. The output from this routine is shown below.

```
Chains:
ID          |  Flags     |  Phase     |  Cycle   |  Nodes
   23912    |  -O-----   |      100   |       0  |  [0.000000]--(100)-->
      10    |  ---LPC-   |        0   |    2399  |  [0.000000]--(100)-->
       1    |  I--L---   |        0   |    2400  |  [0.000000]--(100)-->
```

At the end of the run period, there are three chains: firstly, the original input chain (ID 1); secondly, a looping, predictive, competing chain that encodes the repeating “0” (ID 10); and thirdly, an open

[5] The default running time is four minutes.

chain that was in the process of being built when the CHAINS exited (ID 23912). The large value of the last chain ID is indicative of the fact that many chains have been created and destroyed during these 240 seconds.

The fields of these output chains can be examined by typing `outchns(1)`, `outchns(2)` or `outchns(3)` at the MATLAB prompt. They can be printed again using `chn_print`.

Providing Run Options to CHAINS

The example above used the default options. Run options can be changed by modifying the fields of the run options structure. The example below changes the run options so that the algorithm runs with a clock from 30 to 60 seconds (instead of 0 to 240 seconds).[6]

```
% Fill in default options to start with
runopt = def_runopt;

% Modify options we want to change (run from 30 s to 60 s)
runopt.stepstart = int32(30000);
runopt.stepend   = int32(60000);
```

Other run options are documented: type `help def_runopt`.

Repeated Calls to CHAINS

The `stepstart` and `stepend` run options are particularly useful in that they permit repeated calls to the CHAINS algorithm. The following long example creates an ABA chain and then runs it for two minutes, divided into four 30-second blocks.

```
% Create an empty chain and set make it open to add nodes
ABAchn = chn_new;
ABAchn = chn_setflags(ABAchn, 'open');

% Add a node containing data 0 semitones and advance 150 ms
ABAchn = chn_addnode(ABAchn, 0);
ABAchn = chn_advance(ABAchn, 150);

% Add a node containing data 8 semitones and advance 150 ms
ABAchn = chn_addnode(ABAchn, 8);
ABAchn = chn_advance(ABAchn, 150);

% Add a node containing data 0 semitones and advance 300 ms
ABAchn = chn_addnode(ABAchn, 0);
ABAchn = chn_advance(ABAchn, 300);

% Make chain looping/input
ABAchn = chn_clearflags(ABAchn, 'open');
ABAchn = chn_setflags(ABAchn, {'loop', 'input'});
```

(continued below)

[6] Note that the simulation time step is 1 ms, as in all our simulations reported in the paper.

```
% Reset chain phase to zero
ABAchn = chn_setphase(ABAchn, 0);

% Set the default options
runopt = def_runopt;

% Run from 0s – 30s; put output in outchns30
runopt.stepstart = int32(0);
runopt.stepend   = int32(30000);
outchns30        = chn_run(ABAchn, runopt);

% Run from 30s – 60s; put output in outchns60
runopt.stepstart = int32(30000);
runopt.stepend   = int32(60000);
outchns60        = chn_run(outchns30, runopt);

% Run from 60s – 90s; put output in outchns90
runopt.stepstart = int32(60000);
runopt.stepend   = int32(90000);
outchns90        = chn_run(outchns60, runopt);

% Run from 90s – 120s; put output in outchns120
runopt.stepstart = int32(90000);
runopt.stepend   = int32(120000);
outchns120       = chn_run(outchns90, runopt);
```

Now the state of the CHAINS algorithm (i.e. a set of chains) at 30, 60, 90 and 120 seconds are stored in `outchns30`, `outchns60`, `outchns90` and `outchns120`, respectively.

4. Chain Dynamics

State Variables of a Chain

An important aspect of CHAINS not yet explored in this introduction is **dynamics**. Only competing chains have dynamical states. The instantaneous dynamical state of a chain is stored in the `dyn` field of the chain structure. The recorded history of the dynamical state of a chain is stored in the field `rec`, which is a matrix. The matrix has seven rows, which respectively record the following quantities:

Row	Description
1	excitation (E)
2	inhibition (I)
3	adaptation (A)
4	noise input (U)
5	success (S)
6	normalization (R)
7	input due to discovery (X)

The columns of the `rec` matrix refer to time steps. The time step when the dynamic state first starts being recorded (i.e., the time step of the first column) is given in the field `steprecord` of the chain.

Simple Example

The following example does the following:

- create a chain consisting of 0 semitones repeating every 150 ms
- run the CHAINS algorithm for 5 seconds
- identify the one competing output chain
- plot its recorded dynamics

```
% Create an empty chain and set it open to add nodes
Achn = chn_new;
Achn = chn_setflags(Achn, 'open');

% Add a node containing data 0 semitones and advance 150 ms
Achn = chn_addnode(Achn, 0);
Achn = chn_advance(Achn, 100);

% Make chain looping/input
Achn = chn_clearflags(Achn, 'open');
Achn = chn_setflags(Achn, {'loop', 'input'});

% Reset chain phase to zero
Achn = chn_setphase(Achn, 0);
```

```
% Set run duration to 5 seconds
runopt           = def_runopt;
runopt.stepstart = int32(0);
runopt.stepend   = int32(5000);

% Run CHAINS using A-chain as input
outchns = chn_run(Achn, runopt);

% Identify the index of the output chain which is competing
compix = find(chn_flags(outchns, 'compete'));

% Stop if no competing chain!
if isempty(compix), error('No competing chain!'); end

% Get the competing chain
compchn = outchns(compix);

% Get the time steps (in seconds) when states were recorded
steprec = double(compchn.steprecord);
tsec    = (steprec + (0:size(compchn.rec, 2)-1)) / 1000;

% Get the excitation (row 1)
row    = 1;
states = compchn.rec(row,:);

% Plot state against time
plot(tsec, states, 'k', 'LineWidth', 1.5);
xlim([0, 5]);
ylim([0, 2]);

% Add labels
xlabel 'Time (sec) '
ylabel 'State'
```

The figure output from this routine has the following appearance.

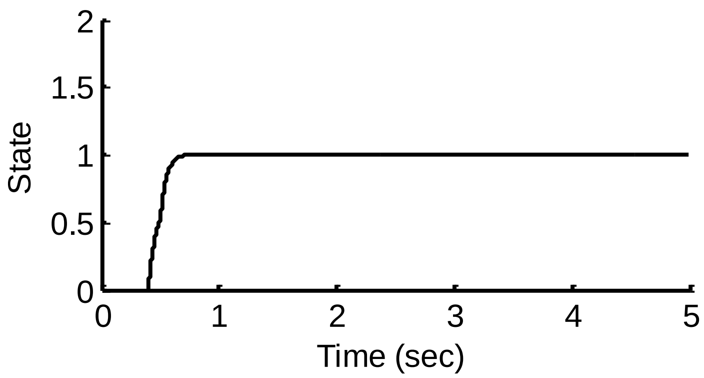

The chain starts competing at 400 ms, and its excitation increases to near the maximum (1) after approximately 200 ms. The chain is fully excited because it has no competitors. The routine above

can be modified to plot other state variables quite easily: one simply plots a different row of the state variable matrix (modify the row shown in bold above) and adjusts the axis limits appropriately.

Associates and State Variables

The state variables E, I, A, U, S, R and X refer to the state of an individual (competing) chain. How to extract these state variables after running the CHAINS algorithm was described above. Because the previous example consisted of a single repeating tone, the only chain in the competition was a repeating tone, with no competitors. When more than one chain can be built from an input sequence and compete with others, things are more complex. This is the case, for example, when an ambiguous ABA input chain is supplied to CHAINS.

Associates

When two chains predict the same event, they become **associates**. Each chain maintains a list of the chains with which it is associated in the structure field `ascs`. Each entry in `ascs` records the unique ID of an associated chain (`ascID`), the time step at which the association was formed (`stepnew`), and other details relating to dynamics between the chains (dyn).

The following example creates an alternating tone, runs the CHAINS algorithm, and identifies the competing chains.

```
% Create an empty chain and set it open to add nodes
ABchn = chn_new;
ABchn = chn_setflags(ABchn, 'open');

% Add a node containing data 0 semitones and advance 100 ms
ABchn = chn_addnode(ABchn, 0);
ABchn = chn_advance(ABchn, 100);

% Add a node containing data 8 semitones and advance another 100 ms
ABchn = chn_addnode(ABchn, 8);
ABchn = chn_advance(ABchn, 100);

% Make chain looping/input
ABchn = chn_clearflags(ABchn, 'open');
ABchn = chn_setflags(ABchn, {'loop', 'input'});

% Reset chain phase to zero
ABchn = chn_setphase(ABchn, 0);

% Run CHAINS using alternating tone as input
outchns = chn_run(ABchn, def_runopt);

% Identify the indices of the output chains which are competing
compixs = find(chn_flags(outchns, 'compete'));

% Stop if no competing chains!
if isempty(compixs), error('No competing chains!'); end
% Get the competing chains
compchns = outchns(compixs);
```

In general, there will be three competing chains that emerge from an alternating (AB) tone: one consisting of As, one consisting of Bs, the other containing A and B. Indeed this is what we see if we print the competing chains using `chn_print(compchns)`:

```
ID        |  Flags    |  Phase    |  Cycle  |  Nodes
    9967  |  ---LPC-  |        0  |    694  |  [0]--(200)-->
    9857  |  ---LPC-  |      100  |    699  |  [8]--(200)-->
      21  |  ---LPC-  |        0  |   1199  |  [0]--(100)-->[8]--(100)-->
```

From this output, however, it is not clear which chain is competing with which. As described above the associates list of each chain answers this question. To see the associations of the chain with ID 21, type[7]

```
ascs21 = [compchns([compchns.ID] == 21).ascs]
```

The chain with ID 21 has two associates, whose IDs can be identified by typing `[ascs21.ascID]`. The IDs of the associates are 9967 and 9857. Following the same procedure for the other two chains, we see that chains 9957 and chain 9967 are associated with chain 21, but the two chains are not associated with each other. In general,

- a chain is not associated with itself
- two chains do not associate, if they never predict the same thing
- association is symmetric (if *x* is an associate of *y*, *y* is an associate of *x*)

Collision State Variables

The collision state variable C_{ij}, tracks the rate of collisions between two chains i and j. Consequently, the C state variable refers to an *association* between two chains, not a single chain; and a C field appears in the dyn field of an association. Similarly, the `rec` field of an association holds a record of the collision between the pair of chains that form the association.

To plot the collision rate between chains 9967 and 21 above, we use the following code.

```
% Get association and time steps
asc  = compchns(1).ascs(1);
tsec = (double(asc.steprecord) + (0:size(asc.rec, 2) - 1)) / 1000;
```

[7] For a different states of the random number generator, the IDs of the chains may vary. Adapt the example to use the appropriate values.

```
% Plot state against time
plot(tsec, asc.rec, 'k', 'LineWidth', 1.5);
xlim([0, 240]);

% Add labels
xlabel 'Time (sec)'
ylabel 'Collision'
```

The output from this example is plotted below. Note that the association forms at about 100 seconds, and the collision rate reaches an asymptote of 5 collisions per second within a few seconds. This reflects the stimulus correctly, because the original sequence was presented with an SOA of 100 ms, and the collisions between the integrated chain and one of the segregated chains occurs on *every other tone*, i.e., 200 ms, or at a rate of 5 per second.

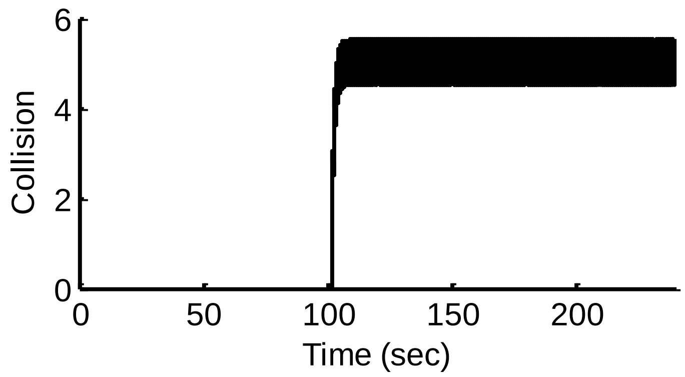

Dynamics Options

The dynamics evolve according to time constants and asymptotic parameters specified in `dynopt` subfield of the run options. These are modified in the same way as the other parameters:

```
% Get default run options
runopt = def_runopt;

% Increase the efficacy of successes upon chain excitation (default 3.8)
runopt.dynopt.alphaS = 4.0;
```

For a description of the dynamics options, type `help def_dynopt`.

A Comprehensive Example

The following code integrates everything that has been discussed so far into a single example. The script can be invoked by typing eg_dyns at the MATLAB command prompt.

eg_dyns.m

```
%% 1. Set the stimulus parameters.

SOA = int32(100); % milliseconds
DF  = 8;          % semitones


%% 2. Create the input ABA chain.

% Create an empty chain and make it open to add nodes
ABAchn = chn_new;
ABAchn = chn_setflags(ABAchn, 'open');

% Add a node containing data 0 semitones and advance 100 ms
ABAchn = chn_addnode(ABAchn, 0);
ABAchn = chn_advance(ABAchn, SOA);

% Add a node containing data 4 semitones and advance 100 ms
ABAchn = chn_addnode(ABAchn, DF);
ABAchn = chn_advance(ABAchn, SOA);

% Add a node containing data 0 semitones and advance 200 ms
ABAchn = chn_addnode(ABAchn, 0);
ABAchn = chn_advance(ABAchn, 2*SOA);

% Make chain looping/input
ABAchn = chn_clearflags(ABAchn, 'open');
ABAchn = chn_setflags(ABAchn, {'loop', 'input'});

% Reset chain phase to zero
ABAchn = chn_setphase(ABAchn, 0);


%% 3. Set the options of the CHAINS algorithm and run it for 240 sec.

% Set options
runopt           = def_runopt;
runopt.stepstart = int32(0);
runopt.stepend   = int32(240*1000);

% Run CHAINS
outchns = chn_run(ABAchn, runopt);
```

(continued below)

```
%% 4. Find and plot all competing chains

% Get chains that are competing
compixs  = find(chn_flags(outchns, 'compete'));
compchns = outchns(compixs);

% Plot competing chains
disp('These are the competing chains:');
chn_print(compchns);


%% 5. Plot the dynamics state variables for individual chains.

% Set the colours to plot each chain in
colours = [1 0 0; 0 0.5 0; 0 0 1];

% Name the state variables
statenames = {'Excitation (E)', 'Inhibition (I)', 'Adaptation (A)', ...
    'Noise (U)', 'Success (S)', 'Normalisation (R)', 'Rediscovery (X)'};

% For each state variable n:
for n = 1:7

    % Create a figure
    figure;

    % For each competing chain m:
    for m = 1:length(compchns)

        % Get the chain, the time steps, and state variable
        chn   = compchns(m);
        tsec  = (double(chn.steprecord) + (0:size(chn.rec, 2) - 1))/1000;
        state = chn.rec(n,:);

        % Plot a line
        line(tsec, state, 'Color', colours(m,:), 'LineWidth', 1.5);

        % Set axis properties
        set(gca, ...
            'XLim', [0 runopt.stepend]/1000, ...
            'LineWidth', 1.5);

        % Add labels/title
        xlabel('Time (s)');
        ylabel(statenames{n});
        title(statenames{n});

    end
```

(continued below)

```
% Add a legend
    IDstrs = arrayfun(@num2str, [compchns.ID], 'UniformOutput', false);
    hl = legend(IDstrs, 'Location', 'NorthEastOutside');
    set(hl, 'Box', 'off');

end


%% 6. Plot the collisions state variables between each competitor.

% Set the colours to plot each chain in
colours = [1 0 0; 0 0.5 0; 0 0 1];

% For each chain n:
for n = 1:length(compchns)

    % Get the associates of chain n
    ascs = compchns(n).ascs;

    % Does this chain have associates?
    if ~isempty(ascs)

        % Create a figure
        figure;

        % For each associate n:
        for m = 1:length(ascs)

            % Get the chain, the time steps, and state variable
            asc   = ascs(m);
            tsec  = (double(asc.steprecord) + (0:size(asc.rec, 2) - 1));
            tsec  = tsec/1000;
            state = asc.rec;

            % Plot a line
            line(tsec, state, 'Color', colours(m,:), 'LineWidth', 1.5);

        end

        % Set axis properties
        set(gca, ...
            'XLim', [0 runopt.stepend]/1000, ...
            'LineWidth', 1.5);

        % Add labels/title
        xlabel('Time (s)');
        ylabel('Collision');
        title(sprintf('Collision rate of ID %d with:', compchns(n).ID));
```
(continued below)

```
% Add a legend
        IDstrs = arrayfun(@(x) ['ID ' num2str(x)], ...
            [ascs.ascID], ...
            'UniformOutput', false);
        hl = legend(IDstrs, 'Location', 'NorthEastOutside');
        set(hl, 'Box', 'off');

    end

end
```

5. Advanced Data Types

One feature of CHAINS is that it can handle discrete sound elements encoded in various ways. By default, the sounds are described by a single, numerical feature, and the **distance** between sound events is the absolute difference in this single feature. (So far, we have been treating this feature as encoding "semitones" on an arbitrarily translated scale.)

CHAINS is able to process sequences of events encapsulating multiple features, but requires a means to compute the distance between sound events. The following sections describe how to set this up.

Symbolic Sounds

CHAINS can process categorical rather than continuous feature values by setting the data type to 'char' (character). This is done by setting the run options as follows:

```
runopt.matlab.type = 'char';
```

Now it is possible to create chains using *characters* as data. By default, the distance between two characters is 0 for identical characters, and 1 for differing characters. This behavior can be overridden however, by supplying the name of a function in the MATLAB workspace that CHAINS should call to compute distance. The function must define a metric, i.e., take two data as arguments and return their distance.

For example, one can write the following function (in an .m file called `mydistance.m`)

```
function d = mydistance(data1, data2)

% Comparing data to itself?
if data1 == data2

    % Zero distance between data and itself
    d = 0;
    return;

end

% Set pairwise distances between distinct data
if data1 == 'A' && data2 == 'B', d = 4; end
if data1 == 'A' && data2 == 'C', d = 5; end
if data1 == 'B' && data2 == 'A', d = 4; end
if data1 == 'B' && data2 == 'C', d = 3; end
if data1 == 'C' && data2 == 'A', d = 5; end
if data1 == 'C' && data2 == 'B', d = 3; end

% end of file %
```

and then instruct CHAINS to use this function to compute distance:

```
runopt.matlab.fn_datadist = 'mydistance';
```

The example code given above sets the perceptual distances between the sounds labeled A, B and C as follows:

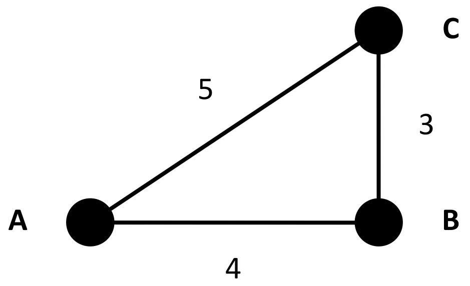

The following example runs a sequence of symbols (alternating B and C) through CHAINS and returns the output chains found as a result.

```
% Create an empty chain and set it open to add nodes
symchn = chn_new;
symchn = chn_setflags(symchn, 'open');

% Add sound event 'B' and advance 100 ms
symchn = chn_addnode(symchn, 'B');
symchn = chn_advance(symchn, 100);

% Add sound event 'C' and advance another 100 ms
symchn = chn_addnode(symchn, 'C');
symchn = chn_advance(symchn, 100);

% Make chain looping/input
symchn = chn_clearflags(symchn, 'open');
symchn = chn_setflags(symchn, {'loop', 'input'});

% Reset chain phase to zero
symchn = chn_setphase(symchn, 0);

% Set run options (char data type with custom distance)
runopt                   = def_runopt;
runopt.matlab.type       = 'char';
runopt.matlab.fn_datadist = 'mydistance';

% Run CHAINS using alternating symbols as input
outchns = chn_run(symchn, runopt);
```

Note that changing the two symbols to A and C will promote segregation, because the distance between A and C (5) is greater than the distance between B and C (3).

Multi-feature Sounds

The scheme by which multi-feature sounds are processed is an extension of the idea given above. Multiple features can be attached to sound events by defining their data type as 'user'. If data types are defined as 'user' then CHAINS can handle any Matlab data type, but the user has to write *two* functions: one to tell CHAINS how to print (one) datum as a string, the other to tell CHAINS how to measure the distance between two data.

For example, these functions might appear as follows.

```
function str = userprint(data)

% Convert data to string
str = [num2str(data.freqHz) 'Hz:' num2str(data.intdB) 'dB'];

% end of file %
```

```
function d = userdist(data1, data2)

% Measure feature-based distance
dfreqHz = (12*(log2(data1.freqHz) - log2(data2.freqHz))).^2;
dintdB  = (data1.intdB - data2.intdB).^2;
d       = 10*sqrt(dfreqHz + dintdB);

% end of file %
```

The run options would then be set as follows.

```
runopt = def_runopt;

runopt.matlab.type         = 'user';
runopt.matlab.fn_datadist  = 'userdist';
runopt.matlab.fn_dataprint = 'userprint';
```

A full example using multi-feature data is given below[8].

```
% Create two multi-feature sounds
A = struct('freqHz', 1000, 'intdB', 40);
B = struct('freqHz', 1200, 'intdB', 50);

% Create an empty chain and set it open to add nodes
chn = chn_new;
chn = chn_setflags(chn, 'open');
```

[8] Note that the distance function and its parameters, as given here, are chosen arbitrarily to illustrate how CHAINS works, and are not intended to match perceptual dynamics. Indeed, finding functions that do match perceptual dynamics is exactly one of the issues that CHAINS can be used to explore.

```
% Add sound event A and advance 100 ms
chn = chn_addnode(chn, A);
chn = chn_advance(chn, 100);

% Add sound event B and advance another 100 ms
chn = chn_addnode(chn, B);
chn = chn_advance(chn, 100);

% Make chain looping/input
chn = chn_clearflags(chn, 'open');
chn = chn_setflags(chn, {'loop', 'input'});

% Reset chain phase to zero
chn = chn_setphase(chn, 0);

% set run options
runopt                      = def_runopt;
runopt.matlab.type          = 'user';
runopt.matlab.fn_dataprint = 'userprint';
runopt.matlab.fn_datadist  = 'userdist';

% Run CHAINS using alternating symbols as input
outchns = chn_run(chn, runopt);
```

The output from CHAINS is as follows:

```
Chains:
ID        |  Flags    |  Phase    |  Cycle  |  Nodes
   33909  |  -O-----  |      100  |      0  |  [1200Hz:50dB]--(100)-->
   33896  |  -O-----  |      200  |      0  |  [1000Hz:40dB]--(200)-->
     776  |  ---LPC-  |        0  |   1190  |  [1000Hz:40dB]--(200)-->
      54  |  ---LPC-  |      100  |   1198  |  [1200Hz:50dB]--(200)-->
      47  |  ---LPC-  |      100  |   1198  |  [1200Hz:50dB]--(100)-->[1000Hz:40dB]--(100)-->
       1  |  I--L---  |        0  |   1200  |  [1000Hz:40dB]--(100)-->[1200Hz:50dB]--(100)-->
```

Note that the custom `userprint` function has been used to print the data in the chain nodes. Increasing the distance between either the frequency or intensity fields of the A and B structures makes the segregated chains easier to form, and the integrated chain harder to form. Conversely, lowering either distance makes the integrated chain easier to form, and the segregated chains harder to form.
